# *7TMRmine*: a Web server for hierarchical mining of 7TMR proteins

**DOI:** 10.1186/1471-2164-10-275

**Published:** 2009-06-19

**Authors:** Guoqing Lu, Zhifang Wang, Alan M Jones, Etsuko N Moriyama

**Affiliations:** 1 Department of Computer Science, University of Nebraska at Omaha, Omaha, NE 68182, USA; 2 Department of Biology, University of Nebraska at Omaha, Omaha, NE 68182, USA; 3 Department of Computer Science and Engineering, University of Nebraska-Lincoln, Lincoln, NE 68588-0660, USA; 4 Departments of Biology, University of North Carolina at Chapel Hill, Chapel Hill, NC 27599, USA; 5 Department of Pharmacology, University of North Carolina at Chapel Hill, Chapel Hill, NC 27599, USA; 6 School of Biological Sciences, University of Nebraska-Lincoln, Lincoln, NE 68588-0118, USA; 7 Center for Plant Science Innovation, University of Nebraska-Lincoln, Lincoln, NE 68588-0118, USA

## Abstract

**Background:**

Seven-transmembrane region-containing receptors (7TMRs) play central roles in eukaryotic signal transduction. Due to their biomedical importance, thorough mining of 7TMRs from diverse genomes has been an active target of bioinformatics and pharmacogenomics research. The need for new and accurate 7TMR/GPCR prediction tools is paramount with the accelerated rate of acquisition of diverse sequence information. Currently available and often used protein classification methods (*e.g*., profile hidden Markov Models) are highly accurate for identifying their membership information among already known 7TMR subfamilies. However, these alignment-based methods are less effective for identifying remote similarities, *e.g*., identifying proteins from highly divergent or possibly new 7TMR families. In this regard, more sensitive (*e.g*., alignment-free) methods are needed to complement the existing protein classification methods. A better strategy would be to combine different classifiers, from more specific to more sensitive methods, to identify a broader spectrum of 7TMR protein candidates.

**Description:**

We developed a Web server, *7TMRmine*, by integrating alignment-free and alignment-based classifiers specifically trained to identify candidate 7TMR proteins as well as transmembrane (TM) prediction methods. This new tool enables researchers to easily assess the distribution of GPCR functionality in diverse genomes or individual newly-discovered proteins. *7TMRmine *is easily customized and facilitates exploratory analysis of diverse genomes. Users can integrate various alignment-based, alignment-free, and TM-prediction methods in any combination and in any hierarchical order. Sixteen classifiers (including two TM-prediction methods) are available on the *7TMRmine *Web server. Not only can the *7TMRmine *tool be used for 7TMR mining, but also for general TM-protein analysis. Users can submit protein sequences for analysis, or explore pre-analyzed results for multiple genomes. The server currently includes prediction results and the summary statistics for 68 genomes.

**Conclusion:**

*7TMRmine *facilitates the discovery of 7TMR proteins. By combining prediction results from different classifiers in a multi-level filtering process, prioritized sets of 7TMR candidates can be obtained for further investigation. *7TMRmine *can be also used as a general TM-protein classifier. Comparisons of TM and 7TMR protein distributions among 68 genomes revealed interesting differences in evolution of these protein families among major eukaryotic phyla.

## Background

Seven-transmembrane-region containing receptors (7TMRs), often referred to as G protein-coupled receptors (GPCRs), constitute the largest receptor superfamily in vertebrates and other metazoans [1-3]. GPCRs, activated by a diverse array of ligands, are the central players in eukaryotic signal transduction and are involved in a wide variety of physiological processes. Mutations in genes encoding GPCRs are associated with major diseases (*e.g*., hypertension, cardiac dysfunction, depression, pain). Due to their biomedical importance, thorough mining of 7TMRs from diverse genomes is an active endeavor of bioinformatics and pharmacogenomics research. However, efforts to identify all member proteins in this superfamily from diverse genomes are hindered by their extreme sequence divergence. In order to facilitate more sensitive and thorough mining, many computational methods, both *alignment-based *and *alignment-free *classification methods, were developed particularly for these proteins.

### Protein classification methods

Computational methods of predicting protein functions rely on detecting similarities among proteins. The majority of protein classification methods rely on alignment to known protein sequences to identify the similarities and to build various forms of models (*e.g*., regular expression patterns [4], protein fingerprints [5], position-specific scoring matrices [6], and profile hidden Markov models [7]). However, generating reliable alignments of divergent candidate 7TMR sequences is practically not possible. Another disadvantage of alignment-based methods is that the resulting models are built only from known "positives" (protein sequences of interest) without incorporating information that discriminates positives from "negatives" (unrelated protein sequences). Consequently, these classifiers are affected by sampling bias, which is propagated and/or amplified during subsequent re-training. In contrast, alignment-free protein classification methods overcome these problems. Instead of alignments, various descriptors are extracted from each sequence (*e.g*., amino acid composition, dipeptide frequencies, and physico-chemical properties), and pattern recognition or multivariate statistical methods are trained to discriminate positive protein samples from negative samples.

Our recent comparative analyses showed that alignment-free classifiers are more sensitive to remote similarities than alignment-based profile hidden Markov model (profile HMM) methods [8-10]. They can also identify weak similarities from short subsequences. We observed also that these alignment-free classifiers are better than profile-HMM methods when a sufficiently large training set is unavailable [9]. For example, one alignment-free method was successfully used to identify extremely divergent 7TMRs (odorant and gustatory receptors) for the first time from the *Drosophila melanogaster *genome [11-13]. One disadvantage of alignment-free classifiers is their relatively high false-positive rate. Profile-HMM classifiers, on the other hand, are accurate in identifying well-established protein family with few false positives. Combining both approaches hierarchically provides greater sensitivity with fewer false positives.

### Hierarchical classification strategy

Our study for mining 7TMR protein candidates from the *Arabidopsis thaliana *genome showed the power of hierarchically combining multiple classifiers, including both traditional alignment-based and newer alignment-free methods [14]. We identified 394 *Arabidopsis thaliana *proteins as 7TMR candidates and selected 54 proteins as those prioritized for further investigation. More recently, Gookin *et al*. [15] used a similar strategy by combining several methods hierarchically and identified a small number of GPCR candidates from three plant genomes including *A. thaliana*. They showed that a subset of the *Arabidopsis *proteins predicted to be GPCR candidates can interact with the *Arabidopsis *G-protein α subunit (AtGPA1) in a yeast complementation assay.

In order to facilitate hierarchical identification of 7TMR proteins, we developed the Web server, *7TMRmine*. *7TMRmine *permits users to customize the integration of both alignment-based and alignment-free classifiers in any combination and order. *7TMRmine *is a Web-based mining system as well as a database for 7TMR candidates from a growing collection of diverse genomes. It allows researchers to generate and explore prioritized lists of 7TMR candidates. It also allows researchers to examine the performance of various methods. Furthermore, *7TMRmine *can be used for other transmembrane protein identification.

### 7TMR proteins

While all known GPCR proteins have seven transmembrane (TM) regions, an increasing number of alternative 'G protein-independent' signaling mechanisms are associated with some 7TM protein groups. For example, plant-specific mildew resistance locus O (MLO) protein family is one of the most divergent 'GPCR' families [16,17], and, not surprisingly, MLO's interaction with Gα has not been shown despite great effort (AM Jones and R Panstruga, unpublished data). Another problem is that none of the candidate plant GPCRs was shown to activate the Gα subunit; therefore they do not fulfill the most important criterion for GPCR classification. A third problem is represented by the odorant receptor (OR) family in insects, another extremely diverged group of 7TM proteins. These proteins act independently of known G-protein-coupled second messenger pathways [18,19]. With these problems acknowledged, it is no longer appropriate to label the entire 7TM protein group as GPCRs because this group includes 'G protein-dependent', 'G protein-independent' signaling proteins, and putative scaffolds. Following the notation used in our previous study [14], we designate these proteins as candidate 7-transmembrane receptors (7TMRs), not GPCRs. Our goal here is to provide a tool capable of identifying the entire set of 7TMRs from diverse genomes. Having a comprehensive inventory of 7TMRs from diverse organisms will facilitate studies on the evolution of GPCRs and to address functionality of the large number of orphaned GPCRs, many critical to human health.

## Construction and content

### Overview of the *7TMRmine *Web server

*7TMRmine *Web server includes protein classifiers and the database of the classification results. The Web interface is developed in HTML, PHP, and PERL. The database is managed in MySQL [20]. The user interface is available through standard Web browsers (tested for Safari, Firefox, and Internet Explorer). The Web server and all classifier programs run on the Linux operating system with the Apache HTTP server (tested on Red Hat Linux 9 and CentOS 4.2/5.1).

**Figure 1:**
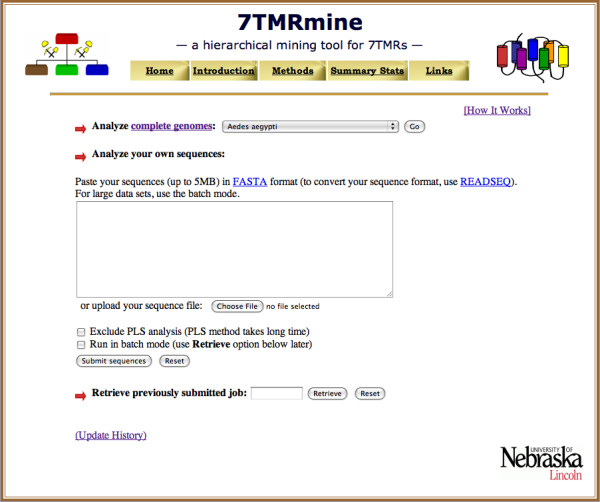
***7TMRmine *Web server**. At the home page, users can choose a genome to explore or submit their own protein sequences for analysis.

**Figure 2:**
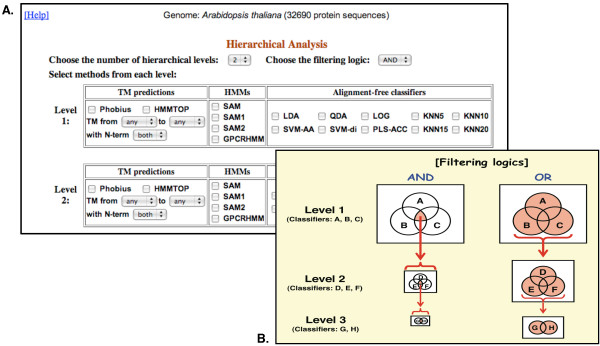
***7TMRmine *hierarchical analysis options**. The hierarchical analysis process is highly customizable (A). The user options include the number of levels, the filtering logic ('AND' or 'OR'), and the combination of classifiers at each level. For the TM predictions, further options are available, including the TM number range and the location of N-terminus ('in' or 'out'). The two filtering logics are used to identify 'positive' proteins (B). 'AND' logic identifies a protein as a positive if all classifiers identify it as a positive (the intersection), otherwise as negatives. 'OR' logic identifies a protein as a positive if at least one classifier identifies it as a positive (the union), otherwise as negatives. Only positively identified proteins are passed to the next level for further analysis.

The database currently includes classification results for 70 complete genomes from 68 different organisms across major eukaryotic phyla (For *A. thaliana*, three versions of genomes, TAIR5, TAIR7, and TAIR8, are included [21,22]). We plan on adding more genomes with regular updates as well as upon user requests. The classification results for user-submitted protein sequences are stored as temporary records in a database table. Figure 1 shows the *7TMRmine *home page where users can either submit their protein sequences in FASTA format or choose from 70 complete genomes to explore. For either option, predictions by different classifiers can be performed individually or hierarchically. For the hierarchical analysis, users can choose the number of hierarchical levels and the combination of classifiers at each level (Figure 2A). Classifier results at each level are combined by using either 'AND' (intersection) or 'OR' (union) logic. As illustrated in Figure 2B, this option lets the users decide how the classifier results are used to filter protein sequences from one level to the next. With the 'AND' logic, the filtering is strict and fewer candidates are submitted to the next level analysis. With the 'OR' logic, the filtering is less strict and more candidates are kept for further analysis. Protein sequences identified as 'positives' at one level are submitted to the next level for further analysis.

### Protein classifiers

Fourteen classifiers (four alignment-based and ten alignment-free) were trained to identify 7TMR candidates and are included in the current *7TMRmine *(Figure 2A):

#### Profile HMM

This is an alignment-based classifier, and provides full probabilistic representation of protein families [*e.g*., [23]]. The program package, Sequence Alignment and Modeling System (SAM, version 3.5) [24,25] is used for implementing profile HMMs. The expect values (E-values) for SAM are calculated based on the constant sample size, 30,000, regardless of the genome size. Therefore, the E-values can be directly compared between different genomes. Strope and Moriyama [10] reported that when the E-value threshold of 0.05 was used, profile-HMM classifiers were highly accurate (nearly 100% accurate) for identifying proteins belonging to the same 7TMR classes (within-class prediction). However, at the same E-value threshold, these classifiers performed much poorly (70% or lower accuracy) in identifying distant 7TMRs (between-class prediction). Therefore, in *7TMRmine*, we chose three E-value thresholds to provide different levels of identification stringency. They are listed as three different classifiers: SAM, SAM1, and SAM2. The SAM classifier uses the most stringent E-value threshold, E = 0.05. The SAM1 classifier uses E = 4.23 as the threshold, which is based on the highest E-value given to *Arabidopsis *MLOs (specifically, MLO3). The SAM2 classifier is the least stringent with the threshold E = 6.52, which is obtained at the minimum error point [26] based on the classification of the training set (total errors: 4 out of 2,030 training samples: no false positive and 4 false negatives).

#### GPCRHMM

This method was developed by Wistrand *et al*. [27]. These authors constructed a compartmentalized HMM incorporating distinct loop length patterns and differences in amino acid composition between cytosolic loops, extracellular loops, and membrane regions based on a diverse set of GPCR sequences. Their training set included eleven of 13 PFAM GPCR protein families [7]. They considered the remaining two divergent families: *Drosophila *odorant receptor family 7tm_6 (PF02949) and the plant family Mlo (PF03094) as the outliers and excluded from their training set. The sensitivity (against 1,706 positives obtained from GPCRDB [28,29]) and false positive rates (against 1,071 negatives) of GPCRHMM are reported as 92.8% and 0–1.18%, respectively [27].

#### LDA, QDA, LOG, and KNN

These classifiers are parametric and non-parametric discrimination methods (linear, quadratic, and logistic discriminant analyses, as well as nonparametric K-nearest neighbor) described by Moriyama and Kim [8]. These classifiers use amino acid composition and physico-chemical properties as sequence descriptors. For KNN classifiers, the number of neighbors, K, is chosen from 5, 10, 15, or 20 and the classifiers are designated KNN5, KNN10, KNN15, and KNN20, respectively. Based on the training set including 1,000 positives (obtained from GPCRDB) and 750 negatives, cross-validation tests showed that these methods have 97.7–98.7% and 2.9–3.6% of true and false positive rates, respectively [8]. S-PLUS statistical package version 8.1.1 for Linux (TIBCO Software Inc., Palo Alto, CA, USA) is used for the classifier development and application.

#### SVM-AA and SVM-di

These are the classifiers based on support vector machines (SVMs), learning machines that make binary classifications based on a hyperplane separating a remapped instance space [30]. Amino acid composition (SVM-AA) and dipeptide frequencies (SVM-di) are used as the sequence descriptors. Strope and Moriyama [10] reported that the true and false positive rates by SVM-AA are >96% and 4–6%, respectively. SVM-AA performed much better than profile-HMM classifier for identifying distant 7TMRs (~90% accuracy by SVM-AA, while lower than 80% by profile HMMs), and similar accuracies were observed with SVM-AA even for short sub-sequences. Bhasin and Raghava [31] used SVM-di for their GPCRpred classifier and showed that 99.5% accuracy from cross-validation tests based on the training set including the five major 7TMR classes. We use SVMlight version 6.01 developed by Joachims [32,33] for the SVM implementation with the radial basis (rbf) kernel function. We performed the grid analysis with five-fold cross validation to obtain the optimal set of parameters (γ for the rbf kernel and the trade-off, C) for our training set. For SVM-AA and SVM-di, the values used were (γ, C) = (155, 0.5) and (417, 0.5291), respectively.

#### PLS-ACC

This classifier uses the partial least squares regression (PLS) with sequence descriptors based on the auto/cross-covariance transformation of amino acid properties [9]. We use an R implementation [34,35]: the PLS package (ver. 2.1-0) developed by Mevik and Wehrens [36,37]. The classification was done using the threshold score, 0.4982, which was obtained at the minimum error point [26]. PLS-ACC was found to perform better than profile-HMM classifiers and PSI-blast when training sets are small and also against short sub-sequences, constantly better than 90% accuracy whereas profile-HMM classifiers fluctuates as low as 80% accuracy [9].

All classifiers except for GPCRHMM were trained using the dataset including 1,015 each of positive (GPCR) and negative (non-GPCR) sequences (these sequences are available on the *7TMRmine *website). GPCR sequences were randomly sampled from GPCRDB (June 2006 release) [28,29]. Only non-GPCR "Class Z (Archaeal/bacterial/fungal opsins)" sequences were excluded from sampling. Non-GPCR sequences were randomly sampled from UniProtKB/SwissProt (manually curated part of UniProt) [38,39]. We manually examined this random-negative set to ensure that no known GPCR sequences were included.

#### Classifier performance against known proteins

In order to understand how these classifiers perform for the actual 7TMR proteins, we tested them against the entire set of sequences obtained from GPCRDB [28,29]. In Additional file 1, the percentage of positives identified by each classifier is summarized. GPCRDB includes one non-GPCR class, "Class Z: Archael/bacterial/fungal opsins", which includes bacteriorhodopsins, proteorhodopsins, and related fungal opsins. They are light-driven proton and chloride pumps. Although these proteins have 7TM regions, they are not GPCRs and not involved with signal transduction. Therefore, we consider these proteins as important negative test samples.

As shown in Additional file 1, the percentage of positives obtained by classifiers varies depending on the GPCR class. Only Class A (Rhodopsin-like), frizzled/smoothened, and vertebrate taste receptors (T2R) are consistently identified at higher than 96% by any classifier. GPCRHMM completely missed insect odorant receptors and plant MLOs. This is because GPCRHMM is not trained for these proteins as described earlier. Compared to alignment-based classifiers (SAM/SAM1/SAM2 and GPCRHMM), all alignment-free classifiers showed very high false positive rates (shown as % positives against Class Z). In order to reduce false positive rates, Moriyama *et al*. [14] took the intersection of six selected classifiers (SVM-AA, SVM-di, PLS-ACC, LDA, QDA, and KNN20). As shown in Additional file 1, this strategy (called "6 class") reduced the false positive rate to ~6% without affecting the true positive rates. By taking the union of "6 class" and GPCRHMM as well as SAM2, we achieved the highest coverage for all GPCR classes without increasing the false positive rate. Additional file 1 also shows the classifier performance against the GPCR datasets from two organisms (*Homo sapiens *and *D. melanogaster*). Using the combination classifier "6 class + GPCRHMM + SAM2", nearly 100% of all known 7TMRs were recovered from these two genomes.

### Transmembrane prediction methods

HMMTOP2.1 [40-42] and TMHMM2.0 [43] are both HMM-based TM-prediction methods. Both are considered to be the two best TM-prediction methods [*e.g*., [44,45]]. Many secreted proteins contain short N-terminal signal peptides, which often have strongly hydrophobic segments; consequently many TM-prediction methods misidentify these signal peptides as TM regions. Phobius [46,47] addressed this problem by combining a signal peptide model, SignalP-HMM [48], and TMHMM improving overall accuracy in detecting and differentiating proteins with signal peptides and proteins with TM segments.

We incorporated HMMTOP2.1 and Phobius in our classifier set. As shown in Figure 2A, users can set their own rules with the number of TM regions (from 0 to 15 or more) and the location of N-terminals (internal or external of cells). Proteins that satisfy these rules are identified as 'positives', and all others 'negatives'. These options give the users flexibility in mining transmembrane proteins. The topology of canonical GPCR proteins has seven TM-regions and the N-terminus located extracellularly. However, no single TM-prediction method predicts exactly seven TM-regions from all known 7TMRs. Among known GPCR sequences in the GPCRDB, less than 85% are predicted to have exactly seven TM-regions by either Phobius or HMMTOP2.1 (Additional file 2; also see [14]). Choosing the TM number ranging from five to nine, for example, covered 99% of the known GPCRs. In addition to the prediction accuracy problem, some divergent 7TMRs may have their N-termini located intracellularly (Additional file 2; also see [49,50]). Furthermore, test sequences may include partial proteins. Therefore, users are advised to use a range in the number of predicted TM regions for identification purpose.

Genes encoding transmembrane proteins constitute 20–30% of both prokaryotic and eukaryotic genomes [51-54]. Therefore, TM-region prediction is in general one of the most important steps for analyzing proteins. Inclusion of TM-prediction options adds flexibility to explore beyond just 7TM proteins. For this purpose, the users may elect to use only TM-prediction options with any number of levels (Figure 2A). In this regard, *7TMRmine *works as a flexible analysis tool for examining TM protein candidates from entire genomes.

### User submitted sequences

For user-submitted protein sequences, all classifiers are run first and the identification results are displayed for users to review. If the user chooses to perform further hierarchical analysis, the option interface similar to Figure 2A is presented, allowing the user to build and perform their own hierarchical 7TMR mining for any sequences.

## Utility and discussion

### 7TMR protein mining from the *Arabidopsis thaliana *genome

7TMR proteins form the largest receptor superfamily in vertebrates and other metazoans (*e.g*., ~800 in human, ~1,000 in *Caenorhabditis elegans*) [29]. However, few 7TMR candidates are reported in plants and fungi. Only 22 candidate *Arabidopsis *7TMRs were described to date [55] (more recent review is found in Moriyama and Opiyo, in press 65). We explored the possibility of finding more divergent groups of 7TMR candidates from the *A. thaliana *genome using both alignment-free and alignment-based methods [14]. For the *7TMRmine *server, we updated all classifiers using a larger training dataset, and added new classifiers (SAM1, SAM2, GPCRHMM, and Phobius). The server also includes a newer release of the *A. thaliana *genome (TAIR8; 32,690 proteins excluding those shorter than 35 amino acids; 27,066 proteins further excluding predicted alternative-splicing products).

Table 1 summarizes the results obtained from the classifiers based on profile HMMs and TM-prediction methods. GPCRHMM predicted 39 proteins (46 including predicted alternative-splicing products) as 7TMR candidates. In *A. thaliana*, currently 22 (27 including predicted alternative-splicing products) are known to be 7TMRs: 15 MLOs (19 including predicted alternative-splicing products), G-protein-coupled receptor 1 (GCR1), *Arabidopsis thaliana *regulator of G-protein signaling 1 (AtRGS1), and five heptahelical transmembrane proteins (HHPs; 6 including predicted alternative-splicing products). GCR1 and AtRGS1 are known to directly interact with the plant Gα subunit GPA1 [56]. AtRGS1 is a putative membrane receptor for D-glucose and also functions as a GTPase activating protein to AtGPA1 [57]. Two proteins, GTG1 and GTG2 (four proteins including predicted alternative-splicing products; [58]), were claimed to be plant GPCRs based on co-immunoprecipitation of AtGPA1 with these membrane proteins. However, GTG1/GTG2 are treated separately here as their animal homologues are reported to be likely channel proteins with no topological similarity to GPCRs [59]. Of the 22 known 7TMR proteins in *A. thaliana*, GPCRHMM recognized only GCR1 as a candidate. The AtRGS1 protein contains the RGS domain (120 amino acids) attached to the 7-TM region. As described also by Gookin *et al.*[15], GPCRHMM does not recognize AtRGS1 as a 7TMR protein unless the C-terminal RGS domain is removed. As expected, none of the MLOs and HHPs was identified by GPCRHMM. As mentioned before, the training dataset used for GPCRHMM excluded any such extremely diverged proteins [27]. On the other hand, the SAM classifiers were trained using the dataset that included wider ranges of 7TMR proteins. Thus both SAM1 and SAM2 identified all 15 MLOs (19 including alternative-splicing products) as well as GCR1 correctly. However, even after removing the RGS domain sequence, SAM classifiers could not identify AtRGS1 positively; only GCR1 was identified positively by both SAM2 and GPCRHMM.

**Table 1:** Number of 7TMR candidates predicted from 27,066 *A. thaliana *proteins.^a^

Classifiers	Number of 7TMR candidates
GPCRHMM	39 (1)^b ^[46]
SAM (E = 0.05)	10 (10) [12]
SAM1 (E = 4.23)	24 (16) [28]
SAM2 (E = 6.52)	28 (16) [32]
	
Phobius: 5–10TM	1,123 (22)* [1,393]
Phobius: 7TM	191 (20) [245]
HMMTOP: 5–10TM	1,207 (22)* [1,499]
HMMTOP: 7TM	197 (13) [252]
	
Phobius & HMMTOP: 5–10TM	969 (22)* [1,212]
Phobius & HMMTOP: 7TM	103 (11) [134]

^a^Protein numbers exclude predicted alternative-splicing products. The total number of proteins in the *A. thaliana *genome is 32,690 including all predicted products. Numbers in square brackets include predicted alternative-splice products.

^b^Numbers in parentheses are those positively identified from the 22 known *A. thaliana *7TMR proteins. * indicates that GTG1 and GTG2 are also identified.

By using either Phobius or HMMTOP, ~200 of 27,066 *A. thaliana *proteins (or ~250 of 32,690 including alternative-splicing products) were predicted to have exactly seven TM-regions. 103 proteins (134 including alternative-splicing products) were predicted to be 7-TM proteins by both methods. The 22 (or 27 including alternative-splicing products) known *A. thaliana *7TMR proteins were predicted to have between six and eight and between seven and ten TM-regions by Phobius and HMMTOP, respectively. Only 11 of the 22 proteins (or 13 of 27 including alternative-splicing products) are predicted to have exactly seven TM-regions by the both methods. Note that GTG1 and GTG2 are predicted to have eight or nine TM-regions (one of the two GTG2 alternative-splicing products, AT4G27630.1, is predicted to have only five TM-regions by both methods). Of the 27,066 *A. thaliana *proteins, 969 proteins have between five and ten TM-regions by both methods. The range "5–10TMs" (by HMMTOP) was also used by Moriyama *et al*. [14] as the best coverage against the entire GPCR dataset for the hierarchical classification.

**Figure 3:**
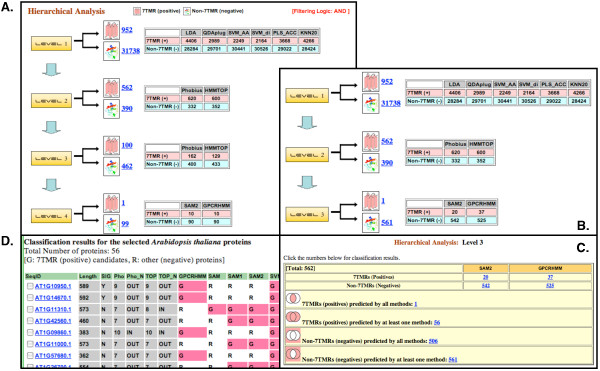
**An example of the hierarchical classification process**. Four hierarchical levels are constructed to analyze the *Arabidopsis thaliana *genome (A). Classifiers included are: six alignment-free classifiers at Level 1, 5–10TMs by both Phobius and HMMTOP at Level 2, 7TMs by both Phobius and HMMTOP at Level 3, and SAM2 and GPCRHMM at Level 4. Less stringent filtering can be done by removing the requirement of 7TMs by Phobius and HMMTOP (B). At each level, prediction results can be combined in various ways (C). In the table listing classification results for the *A. thaliana *genome, each sequence ID is linked to the corresponding gene entry of The Arabidopsis Information Resource (TAIR) website [21,22] (D).

Figure 3 shows an example of hierarchical classification of the *A. thaliana *genome. Four hierarchical levels were generated (Figure 3A). The first level included six alignment-free classifiers chosen in our previous study [14] ("6 class" in Additional file 1). Taking the intersection of all these classifier results ('AND' logic), 952 proteins were identified as 7TMR candidates (positives). At the second level, both TM methods were chosen with the options for 5–10TMs (with no N-terminal preference). Among the 952 proteins identified at the first level, 562 proteins remained as positive. Application of more strict options, seven TMs by the both methods, yielded 100 7TMR candidates at the third level. When SAM2 and GPCRHMM options were used for the final level, only 10 proteins were identified as positives by each of these methods. As shown in Table 1, as few as 50% of currently known *A. thaliana *7TMRs are predicted to have exactly seven TM-regions. Therefore, the requirement of having exactly seven TM-regions seems to be excessively strict. Removing this requirement (Figure 3B), SAM2 identified 20 positives, which included all known MLOs and GCR1. GPCRHMM, on the other hand, identified 37 positives, including only one known 7TMR (GCR1). The positive set predicted by either SAM2 or GPCRHMM (the union set) included 56 proteins (Figure 3C). One can easily change the level-2 options to restrict TM ranges. For example, using 6–10 TMs gave 487 positives with no effect on the SAM2 and GPCRHMM results (20 and 37 positives, respectively). With 7–8 TMs, 156 (132 after excluding alternative transcripts) proteins were identified (see Additional file 3 for the list). This list included all of the 16 high-ranking 7TMR candidates reported by Gookin *et al*. [15] as well as 15 of the 22 known 7TMRs (or 18 of 27 including predicted splicing alternatives). Seven known 7TMRs (6 MLOs and 1 HHP; or nine including predicted splicing-alternatives) were excluded from this list because their number of TM regions did not fit within the chosen range. Both of GTG1 and GTG2 (including all four predicted splicing alternatives) were not included in this list since either or both TM-prediction methods predicted eight or nine TM-regions in GTG1 and GTG2 (one splice form of GTG2 has only five TM-regions). However, GTG1 was positively identified by the all six classifiers, and can be identified as a 7TMR candidate if we relax the TM-number requirement to be between 7 and 9.

As shown in this example, users can choose classifiers in any combination in any number of levels (currently up to six) to create their own hierarchical filtering system. By using less strict methods at the earlier level and more strict methods at the later level, the *7TMRmine *Web server facilitates the prioritization of the 7TMR protein candidate set and generation of a protein set in a manageable size for further investigation. The union and intersection of positive or negative sets can be easily obtained as shown in Figure 3C. Figure 3D shows an example of the list of all classifier prediction results. Protein sequences as well as the classification results can be downloaded from this page for further analysis. For example, protein sequences can be submitted to GPCR classification tools such as GPCRsIdentifier [60], GPCRsclass and GPCRpred [31,61], and GPCRTree [62] for further family classification.

### Distribution of transmembrane proteins among eukaryotic genomes

**Figure 4:**
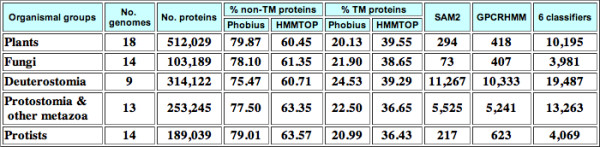
**"TM/7TMR Mining Summary Statistics" page**. Numbers presented are based on the cumulative numbers from each organismal group. "Non-TM proteins" are those predicted to have no (0) TM region. "TM proteins" are those predicted to have at least one (>0) TM regions. The "6 classifiers" column shows the total number of 7TMR candidates predicted by all of LDA, QDA, KNN20, SVM_AA, SVM_di, and PLS_ACC (the intersection of the positives by these classifiers).

**Figure 5:**
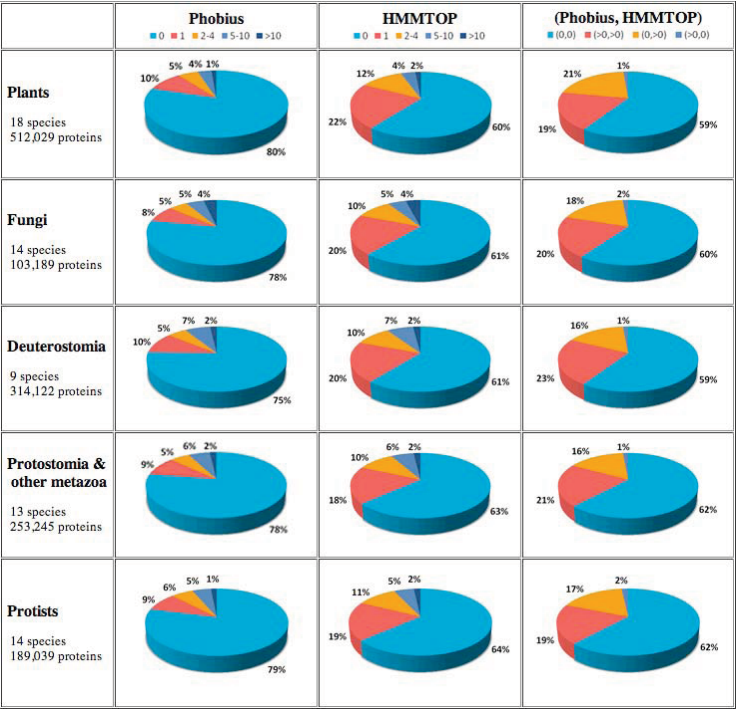
**The "Transmembrane Protein Prediction Statistics" page**. The proportions of proteins predicted to have given numbers of TM regions are illustrated in pie charts for different organismal groups. The "(Phobius, HMMTOP)" column shows the proportions of proteins predicted to have no TM by both methods (0, 0), one or more TMs by both methods (>0, >0), no TM by Phobius but one or more TMs by HMMTOP (0, >0), and one or more TMs by Phobius but no TM by HMMTOP (>0, 0).

**Figure 6:**
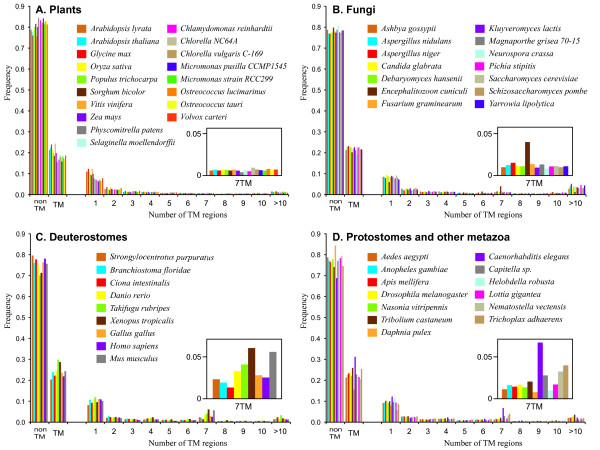
**Transmembrane proteins predicted from four organismal groups**. The histograms compare the frequencies of proteins with different number of TM regions predicted by Phobius among organisms. Proteins predicted to have no (0) TM region are shown above 'nonTM'. Proteins predicted to have one or more (>0) TM regions are shown above 'TM'. The part of the histogram showing the frequencies of 7TM proteins is enlarged and shown in the inset. More histograms are available on the *7TMRmine *website.

Using *7TMRmine*, we examined the distribution of transmembrane proteins among various eukaryotes. The server currently has classification results from 68 organisms across the major eukaryotic phyla: 10 land plants (including 1 moss and 1 fern), 8 green algae, 2 diatoms, 14 fungi, 6 vertebrates, 1 urochordate, 1 cephalochordate, 1 echinoderm, 7 arthropodes, 1 nematode, 2 annelida, 1 mollusca, 1 cnidaria, 1 placozoa, and 11 protists (including 1 red alga, 1 choanoflagellate and 2 *Dictyostelium *species). From each genome, proteins shorter than 35 amino acids and proteins with unidentified residues (irregular letters other than the 20 alphabets, most often 'X') over more than 30% of the length are excluded. The summary statistics are shown in the "TM/7TMR Mining Summary Statistics" page (Figure 4). As mentioned in the earlier section, Phobius predicts fewer TM proteins compared to HMMTOP. The proportion of TM proteins to the entire proteins encoded by the genome was uniform across different organisms, yielding 20–25% by Phobius and ~40% by HMMTOP. In the "Transmembrane Protein Prediction Statistics" page (Figure 5), one can compare the numbers of proteins predicted to have certain numbers of TM regions among different organismal groups. When we compared the TM-prediction results by Phobius with those by HMMTOP, the majority of differences were found in the numbers of 1TM proteins (Figure 5, red) and 2 to 4TM proteins (Figure 5, orange). In all organisms, these two groups of TM proteins were predicted twice more often by HMMTOP than by Phobius, which results in the reduced number of non-TM (0TM) proteins in HMMTOP prediction (Figure 5, light blue). More detailed comparison for each species is presented in histograms (clicking anywhere on the pie charts on the Web page brings the user to the detailed statistics page for the corresponding organism; Figure 6 also shows the histograms only for Phobius prediction). In comparing the histograms of TM numbers predicted by Phobius and HMMTOP, one finds that all of 2-, 3-, and 4-TM proteins are over-presented by HMMTOP, contributing to the increased number of 2–4TM proteins predicted by HMMTOP in Figure 5 (shown with orange). Proteins with higher numbers of TMs also show consistent but much smaller differences between Phobius and HMMTOP. Further examinations showed that among 7,175 *A. thaliana *proteins predicted as non-TM by Phobius and TM by HMMTOP (0, >0), 2,847 proteins (39.7%) were predicted to have signal peptides by Phobius. Among the 18,221 proteins predicted to be non-TM by both methods (0, 0), only 1,177 (6.5%) were predicted to have signal peptides by Phobius. This observation clearly shows that Phobius takes advantage of signal-peptide prediction to avoid misidentifying signal-peptide regions as TM regions. Proteins predicted to have no TM by both methods (0, 0) constitute 60% of any eukaryotic genome; they are most likely truly non-TM proteins. The maximum proportion of non-TM proteins could be ~80% (Figure 5, light blue).

Distributions of TM proteins among four representative organismal groups are compared in Figure 6. While six vertebrates have a greater representation of 7TM proteins among those with multiple TM regions, urochordate (*Ciona intestinalis*) and cephalochordate (*Branchiostoma floridae*) have much smaller numbers of 7TM proteins compared to other vertebrates (Figure 6C). This is consistent with many vertebrates having the largest 7TMR superfamily. Among the other metazoa including protostomes (six insects, *Daphnia pulex*, *C. elegans*, two annelida, one mullusca, as well as *Nematostella vectensis *and *Trichoplax adhaerens*), *C. elegans *shows a significantly higher number of 7TM proteins, the largest among the 68 organisms accounting almost for 7% of its genome (Figure 6D). The majority of these *C. elegans *7TM proteins belong to chemoreceptors [3,63]. It is also interesting to note that two basal metazoa, *N. vectensis *(cnidaria) and *T. adhaerens *(placozoa) have greater representation of 7TM proteins compared to protostomes. On the other hand, plants and protists show no such over-representation of 7TM proteins. Among fungi, there appears to be species-specific over-representation of 7TM proteins in *Encephalitozoon cuniculi*, an animal pathogen with the smallest genome among eukaryotes [64]. Of 1,996 proteins, 91 genes (more than 4% of the genome) are predicted to encode proteins that have seven TM-regions by either Phobius or HMMTOP. Considering that other fungal genomes have only less than 2% (*e.g*., 126 out of 9,838 *Neurospora crassa *proteins) of predicted 7TM proteins and that *E. cuniculi *has reduced gene sets adapted to its parasitic life style, this over-representation of 7TM proteins is significant.

### Distribution of 7TMR proteins among eukaryotic genomes

**Figure 7:**
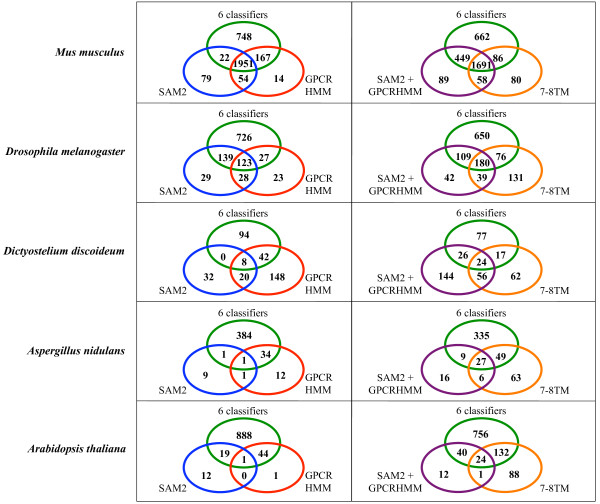
**7TMR protein candidates identified by combination of classifiers**. Venn diagrams show the 7TMR prediction results in various combinations of classifiers. "6 classifiers": the number of 7TMR candidates predicted all of LDA, QDA, KNN20, SVM_AA, SVM_di, and PLS_ACC (the intersection of the positives by these classifiers). "SAM2+GPCRHMM": the number of 7TMR candidates predicted by either of SAM2 or GPCRHMM (the union of the positives by these classifiers). "7–8 TM": the number of proteins predicted to have 7 or 8TMs by both Phobius and HMMTOP. More Venn diagrams are available on the *7TMRmine *website.

The "TM/7TMR Mining Summary Statistics" page also summarizes the distribution of 7TMR protein candidates among eukaryotes (Figure 4). Clearly 7TMR proteins are under-represented in plants, fungi, and protists. For each organismal group, classification results are summarized using Venn diagrams (Figure 7; Venn diagrams for all species are presented on the website). The positives obtained by SAM2 and GPCRHMM have very few overlaps for plant, fungal, and protist proteins (with exception of *D. discoideum*). This result indicates that use of only GPCRHMM, which is not trained for the largest plant 7TMR family (MLO), would omit many 7TMR candidates from these organisms. On the contrary, but as expected, the predictions for deuterostomes by these two classifiers significantly overlap. As described earlier, GPCRHMM is trained to identify canonical GPCRs obtained from these organisms. *C. elegans *of the "protostome" group and *D. discoideum *of the "protist" group show the similar prediction pattern as those for deuterostomes. This is because chemoreceptors from *C. elegans *and cyclic AMP receptors from *D. discoideum*, while divergent, are more closely related to vertebrate types of 7TMRs and GPCRHMM included these sequences for training. On the other hand, insect odorant receptors (ORs) are not included in the training set of GPCRHMM. Therefore, it is not surprising that GPCRHMM does not find the 60 ORs found in *D. melanogaster*. *Drosophila *ORs are included in the 139 proteins recognized by both the 6-classifiers and SAM2 but not by GPCRHMM (Figure 7). Gustatory receptors, similarly divergent insect chemoreceptors, of *D. melanogaster *are also included in this protein set.

### 7TMR candidates in the *A. thaliana*, rice, and poplar genomes

As described earlier, from the *A. thaliana *genome, the 16 high-ranking proteins identified by Gookin *et al*. [15] as well as 15 of the 22 known 7TMRs are found in the 132 proteins (156 including predicted alternative-splice forms) obtained from the intersection of the "6 classifiers" AND "7–8 TM" predictions (see Venn diagrams for *A. thaliana *in Figure 7). All six MLOs of the remaining seven known 7TMRs are included in the 49 proteins (57 including predicted alternative-splice forms) obtained from the intersection between "5–10 TM" AND "SAM2+GPCRHMM" (Venn diagrams including "5–10 TM" are available on the website). The remaining HHP5 as well as GTG1 are predicted as positives by both "5–10 TM" and "6 classifiers" but neither by GPCRHMM nor SAM2. GTG2 is not predicted by "6 classifiers" because PLS-ACC does not identify it as positive. Based on these results, we consider the 162 proteins (excluding predicted alternative-splicing forms; obtained by combining 132 proteins identified by both of "6 classifiers" AND "7–8 TM" with 49 proteins identified by both of "SAM2+GPCRHMM" AND "5–10 TM") to be the most likely 7TMR candidates from the *A. thaliana *genome (see Additional file 3). Similar lists generated for *Oryza sativa *(rice) and *Populus trichocarpa *(California poplar) include 84 and 153 candidates, respectively (see Additional files 4 and 5). High-ranking protein sets identified by Gookin *et al*. [15] included 13 rice and 20 poplar proteins. Of their rice GPCR candidates, six proteins are included in our intersection set of "7–8 TM" AND "6 classifiers", and two proteins are included in the intersection set of "5–10 TM" AND "SAM2+GPCRHMM". Two of the remaining five proteins are included in the intersection set between "5–10 TM" AND "6 classifiers". Three are not identified by any of these criteria due to negative predictions by SVM-AA (for three proteins) and SVM-di (one protein). Among 20 poplar GPCR candidates claimed by Gookin *et al*. [15], 17 proteins are included in our intersection set of "7–8 TM" AND "6 classifiers". Among the three proteins not included in our list, two proteins are predicted to be negatives by SVM-AA.

## Conclusion

*7TMRmine *facilitates the discovery of extremely divergent 7TMR proteins from diverse genomes. By combining prediction results from various classifiers including alignment-based and alignment-free classifiers as well as transmembrane prediction methods in a multi-level filtering process, prioritized sets of 7TMR candidates can be obtained for further investigation. Furthermore, *7TMRmine *can be used as a general transmembrane-protein classifier. Statistics provided for pre-analyzed 68 genomes revealed interesting differences in evolution of these protein families among major eukaryotic phyla.

## Availability and requirements

*7TMRmine *is freely available from  using any current Web browser.

## Authors' contributions

GL wrote part of the programs, carried out analyses of genomes, and revised the manuscript. ZW designed and developed the preliminary version of the database and programs. AMJ contributed to the discussion and writing of the manuscript. ENM conceived of the study, supervised the entire project, wrote part of the programs, carried out analyses of genomes, and wrote the manuscript. ENM also maintains the Web server and database. All authors read and approved the final manuscript.

## Supplementary Material

Additional file 1 **Classifier performance on GPCRDB proteins**. Classifiers were tested against the entire dataset of GPCRDB. The table summarizes the % positive identifications for each GPCR class as well as for two organisms (*Homo sapiens *and *Drosophila melanogaster*). Click here for file

Additional file 2 **Number of transmembrane regions predicted from GPCRDB proteins**. Transmembrane regions were predicted from the entire GPCRDB proteins using two methods, Phobius and HMMTOP. Click here for file

Additional file 3 **7TMR candidate proteins identified from the *Arabidopsis thaliana *genome**. 189 proteins (or 162 proteins excluding predicted alternative-splice products) were obtained by combining the results of eight classifiers and two TM-prediction methods. Click here for file

Additional file 4 **7TMR candidate proteins identified from the *Oryza sativa *genome**. 84 proteins were obtained by combining the results of eight classifiers and two TM-prediction methods. Click here for file

Additional file 5 **7TMR candidate proteins identified from the *Populus trichocarpa *genome**. 153 proteins were obtained by combining the results of eight classifiers and two TM-prediction methods. Click here for file
