# Supplementary material for: 7TMRmine: a Web server for hierarchical mining of 7TMR proteins
Source: BMC Genomics. 2009 Jun 19;10:275. doi: 10.1186/1471-2164-10-275 (PMC2718930; doi:10.1186/1471-2164-10-275)
Supplement: Additional file 1 — Classifier performance on GPCRDB proteins. Classifiers were tested against the entire dataset of GPCRDB. The table summarizes the % positive identifications for each GPCR class as well as for two organisms (Homo sapiens and Drosophila melanogaster). [file 1471-2164-10-275-S1.pdf]

Table S1. Classifier performance on GPCRDB proteins. % positive identifications by each classifier are summarized for each GPCR class as well as for two organisms (*Homo sapiens* and *Drosophila melanogaster*). The highest % positives and those lower than 80% are shown in bold italic and in red color, respectively. For Class Z (non-GPCR), the values indicate % false positives, with higher than 10% in blue color. The last four columns show the results by using combination of classifiers. '6class' is the intersection of SVM-AA, SVM-di, PLS-ACC, LDA, QDA, and KNN20. For other combination classifiers, '+' indicates taking the union and '&' indicates taking the intersection of corresponding classifiers.

	No. proteins	SAM	SAM1	SAM2	GPCRH MM	SVM_ AA	SVM_ di	PLS_ ACC	LDA	QDA	LOG	KNN5	KNN10	KNN15	KNN20	6class	6class + GPCRH MM	6class + GPCRH MM + SAM2	6class & (GPCRH MM + SAM2)
**[GBCRDB class]**																			
**All (excluding Class Z)**	9394	87.27	91.95	92.27	94.07	97.92	99.16	99.49	98.56	97.82	97.64	98.64	98.79	98.81	98.79	95.39	99.18	***99.76***	94.60
**A: Rhodopsin like**	6039	99.95	99.98	99.98	99.14	99.59	99.95	99.98	99.47	99.24	98.77	99.74	99.72	99.70	99.67	98.97	99.95	***100.00***	98.97
**B: Secretin like**	309	98.06	98.71	99.03	96.76	91.26	96.76	88.35	78.64	77.67	77.99	79.29	79.29	80.58	80.58	77.02	97.73	***99.35***	76.70
**C: Metabotropic glutamate / pheromone**	206	65.53	78.16	78.64	93.69	84.95	95.63	80.10	69.90	68.45	64.08	81.07	75.73	69.42	68.93	58.25	93.69	***97.57***	58.25
**D: Fungal pheromone**	65	18.46	20.00	23.08	90.77	93.85	98.46	***100.00***	***100.00***	98.46	98.46	***100.00***	***100.00***	***100.00***	***100.00***	93.85	***100.00***	***100.00***	86.15
**E: cAMP receptors**	10	***100.00***	***100.00***	***100.00***	***100.00***	60.00	80.00	***100.00***	***100.00***	80.00	***100.00***	80.00	80.00	100.00	80.00	60.00	***100.00***	***100.00***	60.00
**Ocular albinism proteins**	8	75.00	***100.00***	***100.00***	***100.00***	***100.00***	***100.00***	***100.00***	***100.00***	***100.00***	***100.00***	***100.00***	***100.00***	***100.00***	***100.00***	***100.00***	***100.00***	***100.00***	***100.00***
**Frizzled/smoothened**	130	97.69	***100.00***	***100.00***	99.23	97.69	***100.00***	98.46	***100.00***	97.69	96.92	97.69	99.23	98.46	96.92	96.15	99.23	***100.00***	96.15
**Insect odorant receptors**	236	89.83	98.73	99.15	0.00	***100.00***	***100.00***	99.15	99.58	99.58	99.58	***100.00***	***100.00***	***100.00***	***100.00***	98.73	98.73	***100.00***	97.88
**Plant Mlo receptors**	52	57.69	82.69	84.62	0.00	92.31	***100.00***	98.08	98.08	98.08	94.23	98.08	***100.00***	***100.00***	***100.00***	90.38	90.38	***98.08***	76.92
**Nematode chemoreceptors**	755	18.68	40.26	42.12	93.51	99.34	98.54	98.54	99.60	99.47	99.47	98.94	99.60	99.60	99.60	96.82	98.01	***98.81***	93.38
**Vomeronasal receptors (V1R&V3R)**	286	56.64	93.01	94.06	98.25	***100.00***	***100.00***	98.60	***100.00***	***100.00***	***100.00***	***100.00***	***100.00***	***100.00***	***100.00***	98.60	***100.00***	***100.00***	97.90
**Taste receptors (T2R)**	237	***100.00***	***100.00***	***100.00***	99.58	0.00	0.00	***100.00***	***100.00***	***100.00***	***100.00***	***100.00***	***100.00***	***100.00***	***100.00***	***100.00***	***100.00***	***100.00***	***100.00***
**Putative**	1061	74.18	83.88	84.54	87.46	90.39	93.69	95.19	92.84	89.44	90.67	92.65	93.78	93.78	93.69	83.79	96.51	***98.77***	81.43
**Z: Archael/bacterial/ fungal opsins (non-GPCR)**	110	0.00	0.00	0.00	0.91	9.09	21.82	91.82	96.36	90.00	96.36	75.45	80.91	90.91	96.36	6.36	6.36	6.36	0.91
**[Species]**																			
Homo sapiens	1426	97.55	98.39	98.39	97.55	97.41	98.81	97.69	95.51	93.76	94.67	96.56	96.21	96.14	96.14	93.06	99.02	***99.79***	92.78
Drosophila melanogaster	314	91.72	93.95	93.95	57.01	93.31	97.45	94.59	89.17	88.54	85.35	94.27	93.95	93.95	92.04	85.03	98.73	***99.68***	85.03
