# Supplementary material for: 7TMRmine: a Web server for hierarchical mining of 7TMR proteins
Source: BMC Genomics. 2009 Jun 19;10:275. doi: 10.1186/1471-2164-10-275 (PMC2718930; doi:10.1186/1471-2164-10-275)
Supplement: Additional file 2 — Number of transmembrane regions predicted from GPCRDB proteins. Transmembrane regions were predicted from the entire GPCRDB proteins using two methods, Phobius and HMMTOP. [file 1471-2164-10-275-S2.pdf]

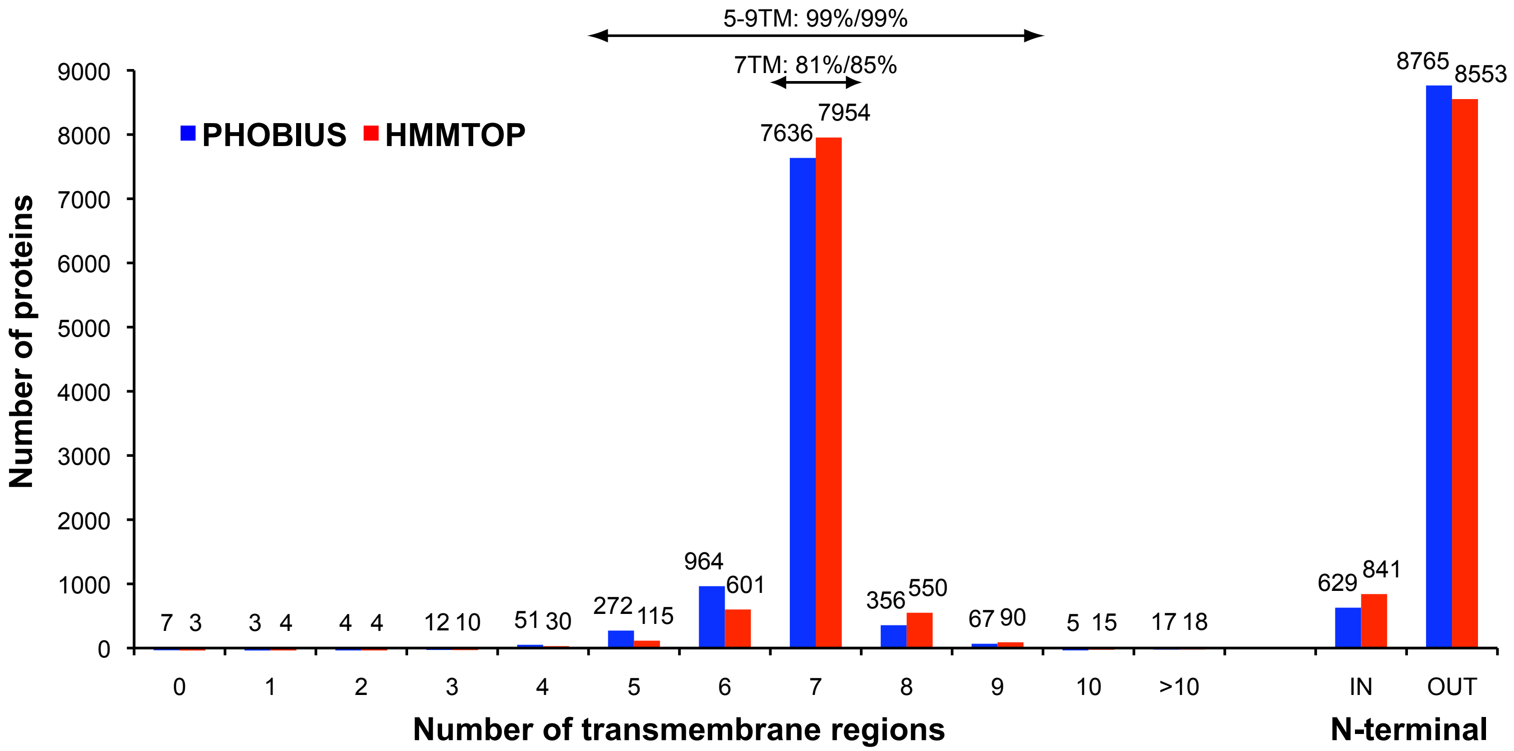

Number of transmembrane regions predicted for different GPCR classes.

GPCR Classes	Proteins	Number of 7TM proteins[a]	
		Phobius	HMMTOP
A: Rhodopsin like	6,039	5,072 (27, 168, 637, 135)	5,427 (24, 202, 353, 33)
B: Secretin like	309	282 (1, 17, 3, 6)	258 (13, 30, 5, 3)
C: Metabotropic glutamate / pheromone	206	190 (2, 4, 5, 5)	145 (10, 37, 8, 6)
D: Fungal pheromone	65	51 (0, 1, 3, 10)	51 (0, 5, 8, 1)
E: cAMP receptors	10	5 (2, 2, 1, 0)	1 (0, 3, 6, 0)
Ocular albinism proteins	8	8 (0, 0, 0, 0)	6 (0, 0, 2, 0)
Frizzled/Smoothened	130	113 (3, 9, 5, 0)	86 (4, 32, 7, 1)
Insect odorant receptors	236	59 (6, 39, 90, 42)	118 (7, 56, 32, 23)
Plant Mlo receptors	52	48 (0, 1, 2, 1)	30 (16, 4, 2, 0)
Nematode chemoreceptors	755	627 (15, 38, 43, 32)	617 (14, 66, 32, 26)
Vomeronasal receptors (V1R & V3R)	286	163 (8, 19, 62, 34)	205 (1, 25, 46, 9)
Taste receptors T2R	237	216 (0, 11, 10, 0)	228 (0, 4, 5, 0)
Putative / unclassified	1,061	802 (25, 47, 103, 84)	782 (34, 86, 95, 64)
Z: Archaeal/bacterial/fungal opsins[b]	110	99 (2, 0, 9, 0)	83 (2, 17, 7, 1)

[a]Number of proteins predicted to have seven TM-regions by Phobius and HMMTOP. Proteins predicted to have “more than 8 TM regions”, “8 TM regions”, “6 TM regions”, and “fewer than 6 TM regions” are shown in parentheses.
[b]Class Z proteins are not GPCRs.

Figure S1. Number of transmembrane regions predicted from GPCRDB proteins. The histogram (top) shows the number of transmembrane regions and the N-terminal location (’IN’ or ‘OUT’ of the cell) predicted from the entire GPCRDB proteins using two methods, Phobius and HMMTOP. The percentages at the top show the proportions of GPCRDB proteins predicted to have the given number of TM regions (Phobius/HMMTOP). The table (bottom) summarizes the number of 7TM proteins predicted by the two methods from different GPCR classes.
