# Supplementary material for: 7TMRmine: a Web server for hierarchical mining of 7TMR proteins
Source: BMC Genomics. 2009 Jun 19;10:275. doi: 10.1186/1471-2164-10-275 (PMC2718930; doi:10.1186/1471-2164-10-275)
Supplement: Additional file 3 — 7TMR candidate proteins identified from the Arabidopsis thaliana genome. 189 proteins (or 162 proteins excluding predicted alternative-splice products) were obtained by combining the results of eight classifiers and two TM-prediction methods. [file 1471-2164-10-275-S3.pdf]

**Table S2. 7TMR candidate proteins identified from the *Arabidopsis thaliana* genome.** 189 proteins (or 162 proteins excluding predicted alternative splice forms) were obtained by combining the results of eight classifiers and two TM prediction methods. 156 proteins (listed with yellow background) were identified by taking the intersection of positives predicted by "6 classifiers" and "7-8 TM". Another 33 proteins (listed with white background) were identified by taking the intersection of positives predicted by "SAM2+GPCRHMM" and "5-10 TM". Proteins represented with more than one alternative transcripts are shown with italics.

**"7TMR":** Names of known 7TMR proteins.
**"Gookin":** Names of the 16 high-ranking GPCR proteins identified by Gookin et al. (2008).
**"Phobius_SIG":** Signal peptide prediction by Phobius (Y: yes, N: no).
**"Phobius":** Number of transmembrane regions predicted by Phobius.
**"Phobius_Nterm":** Location of N-terminus predicted by Phobius (IN: intracellular, OUT: extracellular).
**"HMMTOP":** Number of transmembrane regions predicted by HMMTOP.
**"HMMTOP_Nterm":** Location of N-terminus predicted by HMMTOP (IN: intracellular, OUT: extracellular).
**"GPCRHMM":** GPCR prediction by GPCRHMM (G: GPCR, N: non-GPCR).

| SeqID | 7TMR | Gookin | Length (aa) | Phobius_SIG | Phobius | Phobius_Nterm | HMMTOP | HMMTOP_Nterm | GPCRHMM | Description |
|---|---|---|---|---|---|---|---|---|---|---|
| **AT1G01070.2** | | | 318 | N | 8 | IN | 8 | IN | N | \| Symbols:  \| nodulin MtN21 family protein \| chr1:38898-40597 REVERSE |
| **AT1G03070.1** | | | 247 | N | 7 | OUT | 7 | OUT | N | \| Symbols:  \| glutamate binding / \| chr1:730148-731379 FORWARD |
| **AT1G08350.2** | | | 589 | Y | 9 | OUT | 10 | IN | G | \| Symbols:  \| endomembrane protein 70 family protein \| chr1:2632967-2635602 REVERSE |
| **AT1G09860.1** | | | 383 | N | 10 | IN | 10 | IN | G | \| Symbols: ATPUP16 \| ATPUP16 (Arabidopsis thaliana purine permease 16); purine transmembrane transporter \| chr1:3204138-3205289 REVERSE |
| **AT1G10660.1** | | | 320 | N | 7 | OUT | 7 | OUT | N | \| Symbols:  \| similar to unknown protein [Arabidopsis thaliana] (TAIR:AT5G62960.1); similar to unnamed protein product [Vitis vinifera] (GB:CAO64308.1) \| chr1:3533010-3534782 FORWARD |
| *AT1G10660.2* | | | *320* | *N* | *7* | *OUT* | *7* | *OUT* | *N* | *\| Symbols:  \| similar to unknown protein [Arabidopsis thaliana] (TAIR:AT5G62960.1); similar to unnamed protein product [Vitis vinifera] (GB:CAO64308.1) \| chr1:3533010-3534782 FORWARD* |
| *AT1G10660.3* | | | *320* | *N* | *7* | *OUT* | *7* | *OUT* | *N* | *\| Symbols:  \| similar to unknown protein [Arabidopsis thaliana] (TAIR:AT5G62960.1); similar to unnamed protein product [Vitis vinifera] (GB:CAO64308.1) \| chr1:3533010-3534782 FORWARD* |
| *AT1G10660.4* | | | *320* | *N* | *7* | *OUT* | *7* | *OUT* | *N* | *\| Symbols:  \| similar to unknown protein [Arabidopsis thaliana] (TAIR:AT5G62960.1); similar to unnamed protein product [Vitis vinifera] (GB:CAO64308.1) \| chr1:3533010-3534782 FORWARD* |
| **AT1G10950.1** | | | 589 | Y | 9 | OUT | 9 | OUT | G | \| Symbols:  \| endomembrane protein 70, putative \| chr1:3659322-3663622 FORWARD |
| **AT1G10980.1** | | | 516 | Y | 7 | OUT | 7 | OUT | N | \| Symbols:  \| similar to unknown protein [Arabidopsis thaliana] (TAIR:AT1G61670.1); similar to unnamed protein product [Vitis vinifera] (GB:CAO41520.1); contains InterPro domain Transmembrane receptor, eukaryota; (InterPro:IPR009637) \| chr1:3667531-3669821 REVERSE |
| **AT1G11000.1** | **MLO4** | | 573 | N | 7 | OUT | 7 | OUT | N | \| Symbols: ATMLO4, MLO4 \| MLO4 (MILDEW RESISTANCE LOCUS O 4); calmodulin binding \| chr1:3671935-3675909 REVERSE |
| **AT1G11200.1** | | | 295 | N | 7 | OUT | 7 | OUT | N | \| Symbols:  \| similar to unknown protein [Arabidopsis thaliana] (TAIR:AT4G21570.1); similar to hypothetical protein [Vitis vinifera] (GB:CAN61223.1); contains InterPro domain Protein of unknown function DUF300 (InterPro:IPR005178) \| chr1:3753896-3755459 FORWARD |
| **AT1G11310.1** | **MLO2** | | 573 | N | 7 | OUT | 8 | IN | N | \| Symbols: ATMLO2, PMR2, MLO2 \| MLO2 (MILDEW RESISTANCE LOCUS O 2); calmodulin binding \| chr1:3800899-3803870 REVERSE |
| *AT1G11310.2* | *MLO2* | | *418* | *N* | *6* | *OUT* | *7* | *IN* | *R* | *\| Symbols: ATMLO2, PMR2, MLO2 \| MLO2 (MILDEW RESISTANCE LOCUS O 2) \| chr1:3801546-3803870 REVERSE* |
| **AT1G11450.1** | | | 260 | N | 7 | OUT | 7 | OUT | N | \| Symbols:  \| nodulin MtN21 family protein \| chr1:3854005-3855259 FORWARD |
| *AT1G11450.2* | | | *301* | *N* | *8* | *IN* | *8* | *IN* | *N* | *\| Symbols:  \| nodulin MtN21 family protein \| chr1:3853470-3855259 FORWARD* |
| **AT1G12730.2** | | | 390 | N | 8 | OUT | 8 | OUT | N | \| Symbols:  \| cell division cycle protein-related \| chr1:4335107-4337607 FORWARD |
| **AT1G12750.1** | | | 307 | N | 7 | IN | 7 | IN | N | \| Symbols:  \| rhomboid family protein \| chr1:4345098-4346686 REVERSE |
| *AT1G12750.2* | | | *307* | *N* | *7* | *IN* | *7* | *IN* | *N* | *\| Symbols:  \| rhomboid family protein \| chr1:4345098-4346686 REVERSE* |
| **AT1G13560.1** | | | 389 | N | 8 | IN | 8 | IN | N | \| Symbols: AAPT1 \| AAPT1 (AMINOALCOHOLPHOSPHOTRANSFERASE 1); phosphatidyltransferase \| chr1:4638831-4641688 REVERSE |
| *AT1G13560.2* | | | *346* | *N* | *7* | *OUT* | *8* | *IN* | *N* | *\| Symbols: AAPT1 \| AAPT1 (AMINOALCOHOLPHOSPHOTRANSFERASE 1); phosphatidyltransferase \| chr1:4638831-4641560 REVERSE* |
| **AT1G14530.1** | | **THH1** | 293 | N | 7 | OUT | 7 | OUT | N | \| Symbols:  \| (TOM THREE HOMOLOG); virion binding \| chr1:4971415-4973592 REVERSE |
| *AT1G14530.2* | | | *293* | *N* | *7* | *OUT* | *7* | *OUT* | *N* | *\| Symbols:  \| (TOM THREE HOMOLOG); virion binding \| chr1:4971415-4973592 REVERSE* |
| **AT1G14670.1** | | | 592 | Y | 9 | OUT | 9 | OUT | G | \| Symbols:  \| endomembrane protein 70, putative \| chr1:5037664-5040194 FORWARD |
| **AT1G15110.2** | | | 453 | N | 8 | OUT | 8 | OUT | N | \| Symbols:  \| phosphatidyl serine synthase family protein \| chr1:5199589-5201983 FORWARD |
| **AT1G16560.1** | | | 342 | Y | 7 | OUT | 7 | OUT | N | \| Symbols:  \| Per1-like family protein \| chr1:5668938-5670336 FORWARD |
| *AT1G16560.2* | | | *342* | *Y* | *7* | *OUT* | *7* | *OUT* | *N* | *\| Symbols:  \| Per1-like family protein \| chr1:5668938-5670336 FORWARD* |
| *AT1G16560.3* | | | *342* | *Y* | *7* | *OUT* | *7* | *OUT* | *N* | *\| Symbols:  \| Per1-like family protein \| chr1:5668938-5670336 FORWARD* |
| *AT1G16560.4* | | | *287* | *N* | *7* | *OUT* | *7* | *OUT* | *N* | *\| Symbols:  \| Per1-like family protein \| chr1:5669227-5670336 FORWARD* |
| **AT1G18180.1** | | | 305 | N | 7 | OUT | 8 | OUT | N | \| Symbols:  \| oxidoreductase, acting on the CH-CH group of donors \| chr1:6255896-6257730 FORWARD |
| **AT1G18470.2** | | | 423 | N | 8 | IN | 8 | IN | N | \| Symbols:  \| zinc finger (C3HC4-type RING finger) family protein \| chr1:6356965-6360050 REVERSE |
| **AT1G25500.3** | | | 430 | N | 8 | OUT | 8 | OUT | N | \| Symbols:  \| choline transporter-related \| chr1:8955424-8957281 REVERSE |
| **AT1G26700.1** | **MLO14** | | 554 | N | 7 | OUT | 7 | OUT | N | \| Symbols: ATMLO14, MLO14 \| MLO14 (MILDEW RESISTANCE LOCUS O 14); calmodulin binding \| chr1:9228287-9232224 FORWARD |
| **AT1G34500.1** | | | 341 | N | 8 | IN | 8 | OUT | N | \| Symbols:  \| membrane bound O-acyl transferase (MBOAT) family protein / wax synthase-related \| chr1:12611616-12612641 FORWARD |
| **AT1G42560.1** | **MLO9** | | 460 | N | 7 | OUT | 7 | OUT | N | \| Symbols: MLO9, ATMLO9 \| ATMLO9/MLO9 (MILDEW RESISTANCE LOCUS O 9); calmodulin binding \| chr1:15982649-15985768 REVERSE |
| **AT1G47603.1** | | | 393 | N | 10 | IN | 10 | IN | G | \| Symbols: ATPUP19 \| ATPUP19 (Arabidopsis thaliana purine permease 19) \| chr1:17499279-17500550 REVERSE |
| **AT1G48270.1** | **GCR1** | **GCR1** | 326 | N | 7 | OUT | 7 | OUT | G | \| Symbols: GCR1 \| GCR1 (G-PROTEIN-COUPLED RECEPTOR 1) \| chr1:17831982-17833882 REVERSE |
| **AT1G55130.1** | | | 637 | Y | 9 | OUT | 10 | IN | G | \| Symbols:  \| endomembrane protein 70, putative \| chr1:20573321-20575933 FORWARD |
| **AT1G57680.1** | | **Cand1** | 362 | N | 7 | OUT | 7 | OUT | G | \| Symbols:  \| similar to unnamed protein product [Vitis vinifera] (GB:CAO46875.1); contains InterPro domain Uncharacterised conserved protein UCP031277 (InterPro:IPR016971) \| chr1:21366414-21367502 REVERSE |
| *AT1G57680.2* | | | *362* | *N* | *7* | *OUT* | *7* | *OUT* | *G* | *\| Symbols:  \| similar to unnamed protein product [Vitis vinifera] (GB:CAO46875.1); contains InterPro domain Uncharacterised conserved protein UCP031277 (InterPro:IPR016971) \| chr1:21366414-21367502 REVERSE* |
| **AT1G57980.1** | | | 394 | N | 10 | IN | 10 | IN | G | \| Symbols:  \| purine permease-related \| chr1:21443109-21444397 REVERSE |
| **AT1G61560.1** | **MLO6** | | 583 | N | 7 | OUT | 9 | OUT | R | \| Symbols: ATMLO6, MLO6 \| MLO6 (MILDEW RESISTANCE LOCUS O 6); calmodulin binding \| chr1:22712540-22715697 REVERSE |

| | | | | | | | | | | |
|---|---|---|---|---|---|---|---|---|---|---|
| **AT1G62430.1** | | | 421 | N | 7 | OUT | 8 | OUT | N | \| Symbols: ATCDS1 \| ATCDS1 (CDP-diacylglycerol synthase 1); phosphatidate cytidylyltransferase \| chr1:23109939-23112588 REVERSE |
| **AT1G66770.1** | | | 261 | N | 7 | OUT | 7 | OUT | N | \| Symbols: \| nodulin MtN3 family protein \| chr1:24910114-24910899 REVERSE |
| **AT1G67570.1** | | | 456 | N | 8 | IN | 8 | IN | N | \| Symbols: \| similar to unknown protein [Arabidopsis thaliana] (TAIR:AT1G50630.1); similar to unnamed protein product [Vitis vinifera] (GB:CAO46672.1) \| chr1:25328981-25330601 FORWARD |
| **AT1G68820.1** | | | 468 | Y | 7 | OUT | 7 | IN | N | \| Symbols: \| membrane protein, putative \| chr1:25869515-25872463 FORWARD |
| **AT1G69430.1** | | | 350 | N | 7 | IN | 6 | IN | G | \| Symbols: \| similar to unknown protein [Arabidopsis thaliana] (TAIR:AT1G26650.1); similar to unnamed protein product [Vitis vinifera] (GB:CAO68474.1) \| chr1:26101688-26102740 FORWARD |
| **AT1G72590.1** | | | 320 | N | 7 | OUT | 7 | OUT | N | \| Symbols: \| 3-oxo-5-alpha-steroid 4-dehydrogenase family protein / steroid 5-alpha-reductase family protein \| chr1:27339263-27340651 FORWARD |
| **AT1G73650.2** | | | 291 | N | 7 | OUT | 8 | OUT | N | \| Symbols: \| oxidoreductase, acting on the CH-CH group of donors \| chr1:27692210-27693826 REVERSE |
| **AT1G75000.1** | | | 281 | N | 7 | OUT | 7 | OUT | N | \| Symbols: \| GNS1/SUR4 membrane family protein \| chr1:28167311-28168247 FORWARD |
| **AT1G75470.1** | | | 381 | N | 10 | IN | 10 | IN | G | \| Symbols: ATPUP15 \| ATPUP15 (Arabidopsis thaliana purine permease 15); purine transmembrane transporter \| chr1:28333733-28334961 FORWARD |
| **AT1G77860.1** | | | 351 | N | 7 | IN | 7 | IN | N | \| Symbols: KOM \| KOM (KOMPEITO) \| chr1:29287537-29289352 REVERSE |
| **AT2G01070.1** | | | 496 | Y | 7 | OUT | 7 | OUT | N | \| Symbols: \| similar to unknown protein [Arabidopsis thaliana] (TAIR:AT1G72480.1); similar to Os11g0546100 [Oryza sativa (japonica cultivar-group)] (GB:NP_001068063.1); similar to unnamed protein product [Vitis vinifera] (GB:CAO24763.1); similar to hypothetical protein OsJ_032883 [Oryza sativa (japonica cultivar-group)] (GB:EAZ18674.1); contains InterPro domain Transmembrane receptor, eukaryota; (InterPro:IPR009637) \| chr2:75596-77625 FORWARD |
| *AT2G01070.2* | | | *452* | *Y* | *7* | *OUT* | *8* | *IN* | *N* | *\| Symbols: \| similar to unknown protein [Arabidopsis thaliana] (TAIR:AT1G72480.1); similar to unnamed protein product [Vitis vinifera] (GB:CAO24763.1); contains InterPro domain Transmembrane receptor, eukaryota; (InterPro:IPR009637) \| chr2:75596-77300 FORWARD* |
| **AT2G01970.1** | | | 592 | Y | 9 | OUT | 9 | OUT | G | \| Symbols: \| endomembrane protein 70, putative \| chr2:452196-454818 REVERSE |
| **AT2G02180.1** | | **TOM3** | 303 | N | 7 | OUT | 7 | OUT | N | \| Symbols: TOM3 \| TOM3 (tobamovirus multiplication protein 3) \| chr2:560975-562960 FORWARD |
| **AT2G03120.1** | | | 344 | N | 8 | IN | 8 | IN | N | \| Symbols: \| signal peptide peptidase family protein \| chr2:937551-940080 FORWARD |
| **AT2G13650.2** | | | 284 | N | 8 | IN | 8 | IN | N | \| Symbols: GONST1 \| GONST1 (GOLGI NUCLEOTIDE SUGAR TRANSPORTER 1) \| chr2:5695067-5697385 REVERSE |
| **AT2G16530.1** | | | 343 | N | 7 | OUT | 7 | OUT | N | \| Symbols: \| 3-oxo-5-alpha-steroid 4-dehydrogenase family protein / steroid 5-alpha-reductase family protein \| chr2:7170358-7172146 REVERSE |
| *AT2G16530.2* | | | *342* | *N* | *7* | *OUT* | *7* | *OUT* | *N* | *\| Symbols: \| 3-oxo-5-alpha-steroid 4-dehydrogenase family protein / steroid 5-alpha-reductase family protein \| chr2:7170358-7172146 REVERSE* |
| **AT2G17430.1** | **MLO7** | | 542 | Y | 7 | OUT | 10 | IN | R | \| Symbols: ATMLO7, MLO7 \| ATMLO7/MLO7 (MILDEW RESISTANCE LOCUS O 7); calmodulin binding \| chr2:7575100-7577840 FORWARD |
| **AT2G17480.1** | **MLO8** | | 593 | Y | 7 | OUT | 10 | IN | R | \| Symbols: ATMLO8, MLO8 \| MLO8 (MILDEW RESISTANCE LOCUS O 8); calmodulin binding \| chr2:7597641-7600850 REVERSE |
| **AT2G20650.1** | | | 559 | N | 8 | IN | 7 | IN | N | \| Symbols: \| zinc finger (C3HC4-type RING finger) family protein \| chr2:8911035-8914098 REVERSE |
| *AT2G20650.2* | | | *559* | *N* | *8* | *IN* | *7* | *IN* | *N* | *\| Symbols: \| zinc finger (C3HC4-type RING finger) family protein \| chr2:8911035-8914098 REVERSE* |
| **AT2G21080.1** | | | 414 | N | 7 | IN | 7 | IN | N | \| Symbols: \| similar to extracellular ligand-gated ion channel [Arabidopsis thaliana] (TAIR:AT3G20300.1); similar to unknown protein [Arabidopsis thaliana] (TAIR:AT1G50630.1); similar to unnamed protein product [Vitis vinifera] (GB:CAO43972.1) \| chr2:9050788-9052194 FORWARD |
| **AT2G24150.1** | **HHP3** | | 344 | N | 7 | IN | 8 | OUT | N | \| Symbols: HHP3 \| HHP3 (heptahelical protein 3); receptor \| chr2:10272712-10274425 REVERSE |
| **AT2G25540.1** | | | 1065 | N | 8 | OUT | 8 | OUT | R | \| Symbols: CESA10 \| CESA10 (CELLULOSE SYNTHASE 10); transferase, transferring glycosyl groups \| chr2:10874148-10879155 REVERSE |
| **AT2G29050.1** | | | 389 | N | 7 | IN | 7 | IN | N | \| Symbols: ATRBL1 \| ATRBL1 (ARABIDOPSIS THALIANA RHOMBOID-LIKE 1) \| chr2:12485322-12487198 FORWARD |
| *AT2G29050.2* | | | *346* | *N* | *7* | *IN* | *7* | *IN* | *N* | *\| Symbols: ATRBL1 \| ATRBL1 (ARABIDOPSIS THALIANA RHOMBOID-LIKE 1) \| chr2:12485322-12486730 FORWARD* |
| **AT2G29650.2** | | | 398 | N | 8 | IN | 8 | IN | N | \| Symbols: \| inorganic phosphate transporter, putative \| chr2:12681336-12683080 REVERSE |
| **AT2G31440.1** | | | 250 | N | 7 | OUT | 7 | OUT | N | \| Symbols: \| protein binding \| chr2:13406386-13408048 REVERSE |
| **AT2G32540.1** | | | 755 | N | 8 | OUT | 7 | IN | N | \| Symbols: CSLB04, ATCSLB4, ATCSLB04 \| ATCSLB04 (Cellulose synthase-like B4); transferase/ transferase, transferring glycosyl groups \| chr2:13821763-13825366 FORWARD |
| **AT2G32620.1** | | | 757 | N | 8 | OUT | 8 | OUT | N | \| Symbols: CSLB02, ATCSLB2, ATCSLB02 \| ATCSLB02 (Cellulose synthase-like B2); transferase/ transferase, transferring glycosyl groups \| chr2:13847821-13851401 FORWARD |
| **AT2G33670.1** | **MLO5** | | 501 | N | 7 | OUT | 7 | OUT | N | \| Symbols: ATMLO5, MLO5 \| MLO5 (MILDEW RESISTANCE LOCUS O 5); calmodulin binding \| chr2:14252214-14254955 FORWARD |
| **AT2G34980.1** | | | 303 | N | 8 | IN | 8 | IN | N | \| Symbols: SETH1 \| SETH1; transferase \| chr2:14756046-14756957 FORWARD |
| **AT2G36305.1** | | | 311 | N | 8 | OUT | 8 | OUT | N | \| Symbols: \| prenyl-dependent CAAX protease \| chr2:15221686-15223528 FORWARD |
| **AT2G39060.1** | | | 258 | N | 7 | OUT | 7 | OUT | N | \| Symbols: \| nodulin MtN3 family protein \| chr2:16313896-16315284 REVERSE |
| **AT2G39200.1** | **MLO12** | | 576 | N | 7 | OUT | 9 | OUT | R | \| Symbols: ATMLO12, MLO12 \| MLO12 (MILDEW RESISTANCE LOCUS O 12); calmodulin binding \| chr2:16363333-16366875 REVERSE |
| **AT2G41050.1** | | | 376 | N | 7 | OUT | 7 | OUT | N | \| Symbols: \| PQ-loop repeat family protein / transmembrane family protein \| chr2:17130466-17132700 REVERSE |
| **AT2G41610.1** | | | 310 | N | 7 | OUT | 7 | OUT | N | \| Symbols: \| similar to unnamed protein product [Vitis vinifera] (GB:CAO24352.1) \| chr2:17354259-17355191 FORWARD |
| **AT2G41705.1** | | | 461 | N | 9 | IN | 9 | IN | G | \| Symbols: \| camphor resistance CrcB family protein \| chr2:17405161-17406988 FORWARD |
| **AT2G43070.1** | | | 540 | Y | 9 | OUT | 10 | IN | G | \| Symbols: \| protease-associated (PA) domain-containing protein \| chr2:17918310-17921853 REVERSE |
| **AT2G44110.1** | **MLO15** | | 496 | N | 7 | OUT | 7 | OUT | N | \| Symbols: ATMLO15, MLO15 \| MLO15 (MILDEW RESISTANCE LOCUS O 15); calmodulin binding \| chr2:18252565-18255392 REVERSE |
| *AT2G44110.2* | *MLO15* | | *497* | *N* | *7* | *OUT* | *7* | *OUT* | *N* | *\| Symbols: ATMLO15, MLO15 \| MLO15 (MILDEW RESISTANCE LOCUS O 15); calmodulin binding \| chr2:18252565-18255392 REVERSE* |
| **AT2G46060.1** | | | 807 | Y | 7 | OUT | 7 | OUT | N | \| Symbols: \| transmembrane protein-related \| chr2:18948428-18952073 REVERSE |
| **AT2G46890.1** | | | 322 | N | 8 | IN | 8 | IN | N | \| Symbols: \| oxidoreductase, acting on the CH-CH group of donors \| chr2:19273948-19275203 REVERSE |
| **AT2G47115.1** | | | 300 | N | 7 | OUT | 7 | OUT | N | \| Symbols: \| similar to unknown protein [Arabidopsis thaliana] (TAIR:AT1G10660.3); similar to unknown protein [Arabidopsis thaliana] (TAIR:AT1G10660.1); similar to unknown protein [Arabidopsis thaliana] (TAIR:AT1G10660.4); similar to unnamed protein product [Vitis vinifera] (GB:CAO40183.1) \| chr2:19352446-19353934 REVERSE |
| **AT2G47760.3** | | | 380 | N | 8 | IN | 7 | IN | N | \| Symbols: \| ALG3 family protein \| chr2:19573003-19575291 REVERSE |
| **AT3G01550.1** | | | 383 | N | 7 | OUT | 7 | OUT | N | \| Symbols: \| triose phosphate/phosphate translocator, putative \| chr3:216954-218863 REVERSE |
| **AT3G05010.1** | | **Cand2** | 300 | N | 7 | OUT | 7 | OUT | G | \| Symbols: \| transmembrane protein, putative \| chr3:1389609-1391468 FORWARD |
| **AT3G05940.1** | | **Cand10** | 422 | N | 7 | OUT | 7 | OUT | N | \| Symbols: \| similar to unknown protein [Arabidopsis thaliana] (TAIR:AT5G26740.3); similar to unknown protein [Arabidopsis thaliana] (TAIR:AT5G26740.1); similar to unknown protein [Arabidopsis thaliana] (TAIR:AT5G26740.2); similar to unknown [Populus trichocarpa] (GB:ABK95399.1); contains InterPro domain Protein of unknown function DUF300 (InterPro:IPR005178) \| chr3:1777598-1779654 REVERSE |
| **AT3G06460.1** | | | 298 | N | 7 | OUT | 7 | OUT | N | \| Symbols: \| GNS1/SUR4 membrane family protein \| chr3:1982687-1983583 FORWARD |
| **AT3G06470.1** | | | 278 | N | 7 | OUT | 7 | OUT | N | \| Symbols: \| GNS1/SUR4 membrane family protein \| chr3:1984212-1985048 FORWARD |
| **AT3G09570.1** | | | 439 | Y | 7 | OUT | 7 | OUT | G | \| Symbols: \| similar to unknown protein [Arabidopsis thaliana] (TAIR:AT5G18520.1); similar to unnamed protein product [Vitis vinifera] (GB:CAO18079.1); contains InterPro domain Transmembrane receptor, eukaryota; (InterPro:IPR009637) \| chr3:2940529-2941848 FORWARD |
| **AT3G10980.1** | | | 557 | N | 6 | IN | 6 | IN | G | \| Symbols: \| similar to unknown protein [Arabidopsis thaliana] (TAIR:AT5G05350.1); similar to unnamed protein product [Vitis vinifera] (GB:CAO41459.1); contains InterPro domain Protein of unknown function Cys-rich (InterPro:IPR006461) \| chr3:3438815-3440507 FORWARD |
| **AT3G14770.1** | | | 236 | N | 7 | OUT | 7 | OUT | N | \| Symbols: \| nodulin MtN3 family protein \| chr3:4957794-4959209 REVERSE |

| | | | | | | | | | | |
|---|---|---|---|---|---|---|---|---|---|---|
| **AT3G16690.1** | | | 230 | N | 7 | OUT | 7 | OUT | N | \| Symbols:  \| nodulin MtN3 family protein \| chr3:5684569-5686431 REVERSE |
| **AT3G21580.2** | | | 384 | N | 7 | IN | 8 | OUT | N | \| Symbols:  \| cobalt ion transmembrane transporter \| chr3:7602015-7603727 REVERSE |
| **AT3G25805.1** | | | 335 | N | 7 | IN | 7 | IN | N | \| Symbols:  \| similar to unnamed protein product [Vitis vinifera] (GB:CAO42121.1) \| chr3:9427591-9429406 REVERSE |
| **AT3G26090.1** | **AtRGS1** | **AtRGS1** | 459 | N | 7 | OUT | 7 | OUT | N | \| Symbols: ATRGS1, RGS1 \| RGS1 (REGULATOR OF G-PROTEIN SIGNALING 1) \| chr3:9534120-9536647 FORWARD |
| **AT3G27770.1** | | | 315 | N | 7 | OUT | 7 | OUT | N | \| Symbols:  \| similar to unknown protein [Arabidopsis thaliana] (TAIR:AT5G62960.1); similar to unnamed protein product [Vitis vinifera] (GB:CAO14740.1) \| chr3:10287055-10288711 REVERSE |
| *AT3G27770.2* | | | *272* | *N* | *7* | *OUT* | *7* | *OUT* | *N* | *\| Symbols:  \| similar to unknown protein [Arabidopsis thaliana] (TAIR:AT5G62960.1); similar to unnamed protein product [Vitis vinifera] (GB:CAO14740.1) \| chr3:10287055-10288698 REVERSE* |
| **AT3G28007.1** | | | 251 | N | 7 | OUT | 7 | OUT | N | \| Symbols:  \| nodulin MtN3 family protein \| chr3:10409480-10410870 REVERSE |
| **AT3G45290.1** | **MLO3** | | 508 | N | 7 | OUT | 8 | OUT | N | \| Symbols: ATMLO3, MLO3 \| MLO3 (MILDEW RESISTANCE LOCUS O 3); calmodulin binding \| chr3:16628571-16632337 FORWARD |
| **AT3G48740.1** | | | 289 | N | 7 | OUT | 7 | OUT | N | \| Symbols:  \| nodulin MtN3 family protein \| chr3:18063799-18065648 REVERSE |
| **AT3G49840.1** | | | 457 | N | 7 | OUT | 8 | IN | N | \| Symbols:  \| proline-rich family protein \| chr3:18495536-18497672 FORWARD |
| **AT3G53780.2** | | | 394 | N | 7 | IN | 7 | IN | N | \| Symbols:  \| rhomboid family protein \| chr3:19935707-19937626 REVERSE |
| **AT3G54730.1** | | | 387 | N | 7 | IN | 7 | IN | N | \| Symbols:  \| similar to ATOFP9/OFP9 (Arabidopsis thaliana ovate family protein 9) [Arabidopsis thaliana] (TAIR:AT4G04030.1) \| chr3:20270237-20271400 FORWARD |
| **AT3G59090.1** | | **Cand3** | 367 | N | 7 | OUT | 7 | OUT | G | \| Symbols:  \| similar to TOM1 (TOBAMOVIRUS MULTIPLICATION 1) [Arabidopsis thaliana] (TAIR:AT4G21790.1); similar to unnamed protein product [Vitis vinifera] (GB:CAO66926.1) \| chr3:21850311-21853325 FORWARD |
| *AT3G59090.2* | | Cand4 | *373* | *N* | *7* | *OUT* | *7* | *OUT* | *G* | *\| Symbols:  \| similar to TOM1 (TOBAMOVIRUS MULTIPLICATION 1) [Arabidopsis thaliana] (TAIR:AT4G21790.1); similar to unnamed protein product [Vitis vinifera] (GB:CAO66926.1) \| chr3:21850311-21853426 FORWARD* |
| *AT3G59090.3* | | Cand5 | *361* | *N* | *7* | *OUT* | *7* | *OUT* | *G* | *\| Symbols:  \| similar to TOM1 (TOBAMOVIRUS MULTIPLICATION 1) [Arabidopsis thaliana] (TAIR:AT4G21790.1); similar to unnamed protein product [Vitis vinifera] (GB:CAO66926.1) \| chr3:21850357-21853325 FORWARD* |
| **AT3G60590.3** | | | 404 | N | 7 | OUT | 7 | IN | N | \| Symbols:  \| similar to unknown protein [Arabidopsis thaliana] (TAIR:AT1G48460.1); similar to unknown [Populus trichocarpa x Populus deltoides] (GB:ABK96523.1) \| chr3:22409203-22410728 FORWARD |
| **AT3G63310.1** | | | 239 | N | 7 | OUT | 7 | OUT | N | \| Symbols:  \| glutamate binding / \| chr3:23398913-23399857 REVERSE |
| **AT4G01320.1** | | | 424 | N | 7 | OUT | 7 | OUT | N | \| Symbols: STE24, ATSTE24 \| ATSTE24 \| chr4:545905-549002 FORWARD |
| **AT4G01430.2** | | | 252 | N | 7 | OUT | 7 | IN | N | \| Symbols:  \| nodulin MtN21 family protein \| chr4:585707-587691 FORWARD |
| **AT4G02600.1** | **MLO1** | | 526 | N | 7 | OUT | 9 | OUT | R | \| Symbols: MLO1, ATMLO1 \| ATMLO1/MLO1 (MILDEW RESISTANCE LOCUS O 1); calmodulin binding \| chr4:1144141-1147156 FORWARD |
| *AT4G02600.2* | *MLO1* | | *526* | *N* | *7* | *OUT* | *9* | *OUT* | *R* | *\| Symbols: MLO1, ATMLO1 \| ATMLO1/MLO1 (MILDEW RESISTANCE LOCUS O 1); calmodulin binding \| chr4:1144141-1147156 FORWARD* |
| **AT4G02690.1** | | | 248 | N | 7 | OUT | 7 | OUT | N | \| Symbols:  \| glutamate binding / \| chr4:1186023-1187534 FORWARD |
| **AT4G03820.2** | | | 453 | N | 8 | IN | 8 | IN | N | \| Symbols:  \| similar to unknown protein [Arabidopsis thaliana] (TAIR:AT4G22270.1); similar to unnamed protein product [Vitis vinifera] (GB:CAO23242.1) \| chr4:1772161-1774378 REVERSE |
| **AT4G08290.2** | | | 282 | N | 8 | IN | 8 | IN | N | \| Symbols:  \| nodulin MtN21 family protein \| chr4:5239085-5240395 FORWARD |
| **AT4G08700.1** | | | 361 | N | 10 | IN | 10 | IN | G | \| Symbols: ATPUP13 \| ATPUP13 (Arabidopsis thaliana purine permease 13); purine transmembrane transporter \| chr4:5565995-5567283 REVERSE |
| **AT4G10850.1** | | | 258 | N | 7 | OUT | 7 | OUT | N | \| Symbols:  \| nodulin MtN3 family protein \| chr4:6675064-6676714 FORWARD |
| **AT4G13345.1** | | | 394 | N | 10 | OUT | 10 | OUT | G | \| Symbols: MEE55 \| MEE55 (maternal effect embryo arrest 55) \| chr4:7767288-7769422 FORWARD |
| *AT4G13345.2* | | | *394* | *N* | *10* | *OUT* | *10* | *OUT* | *G* | *\| Symbols: MEE55 \| MEE55 (maternal effect embryo arrest 55) \| chr4:7767288-7769422 FORWARD* |
| **AT4G14730.1** | | | 235 | N | 7 | IN | 7 | IN | N | \| Symbols:  \| transmembrane protein-related \| chr4:8448602-8449996 FORWARD |
| **AT4G15290.1** | | | 757 | N | 7 | OUT | 7 | IN | N | \| Symbols: CSLB05, ATCSLB5, ATCSLB05 \| ATCSLB05 (Cellulose synthase-like B5); transferase/ transferase, transferring glycosyl groups \| chr4:8721693-8726599 REVERSE |
| **AT4G15470.1** | | | 256 | N | 7 | IN | 7 | IN | N | \| Symbols:  \| Identical to Protein FBL4 (FBL4) [Arabidopsis Thaliana] (GB:Q94A20;GB:O23399;GB:Q940C4); similar to glutamate binding / [Arabidopsis thaliana] (TAIR:AT1G03070.1); similar to unknown [Populus trichocarpa] (GB:ABK94990.1); contains InterPro domain Protein of unknown function UPF0005 (InterPro:IPR006214) \| chr4:8843659-8845503 FORWARD |
| **AT4G15920.1** | | | 241 | N | 7 | OUT | 7 | OUT | N | \| Symbols:  \| nodulin MtN3 family protein \| chr4:9030756-9033357 REVERSE |
| **AT4G17250.1** | | | 416 | N | 7 | OUT | 8 | OUT | N | \| Symbols:  \| similar to unknown protein [Arabidopsis thaliana] (TAIR:AT5G47580.1); similar to hypothetical protein 40.t00008 [Brassica oleracea] (GB:ABD65133.1) \| chr4:9671116-9672366 FORWARD |
| **AT4G17580.1** | | | 247 | N | 7 | OUT | 7 | OUT | N | \| Symbols:  \| Bax inhibitor-1 family protein / BI-1 family protein \| chr4:9789802-9791158 REVERSE |
| **AT4G18195.1** | | | 394 | N | 10 | IN | 10 | IN | G | \| Symbols: ATPUP8, AT4G18200 \| AT4G18200/ATPUP8 (Arabidopsis thaliana purine permease 8); purine transmembrane transporter \| chr4:10069729-10070985 FORWARD |
| **AT4G18197.1** | | | 390 | N | 10 | IN | 10 | IN | G | \| Symbols: ATPUP7, AT4G18200, PEX17 \| AT4G18200/ATPUP7/PEX17 (ARABIDOPSIS THALIANA PURINE PERMEASE 7); purine transmembrane transporter \| chr4:10071786-10073040 FORWARD |
| **AT4G18205.1** | | | 377 | N | 10 | IN | 10 | IN | G | \| Symbols: AT4G18200 \| AT4G18200 \| chr4:10073861-10075076 FORWARD |
| **AT4G18220.1** | | | 344 | N | 9 | OUT | 9 | OUT | G | \| Symbols:  \| purine permease family protein \| chr4:10078779-10080120 FORWARD |
| **AT4G20100.1** | | | 288 | N | 7 | OUT | 7 | OUT | N | \| Symbols:  \| PQ-loop repeat family protein / transmembrane family protein \| chr4:10874009-10874875 REVERSE |
| **AT4G21790.1** | | **TOM1** | 291 | N | 8 | IN | 7 | OUT | G | \| Symbols: TOM1 \| TOM1 (TOBAMOVIRUS MULTIPLICATION 1) \| chr4:11569936-11572175 FORWARD |
| **AT4G22340.1** | | | 423 | N | 7 | OUT | 8 | OUT | N | \| Symbols:  \| phosphatidate cytidylyltransferase, putative / CDP-diglyceride synthetase, putative \| chr4:11800162-11802561 REVERSE |
| *AT4G22340.3* | | | *447* | *N* | *7* | *OUT* | *8* | *OUT* | *N* | *\| Symbols:  \| phosphatidate cytidylyltransferase, putative / CDP-diglyceride synthetase, putative \| chr4:11800162-11802633 REVERSE* |
| **AT4G23070.1** | | | 313 | N | 7 | OUT | 7 | OUT | N | \| Symbols:  \| rhomboid family protein \| chr4:12090701-12092088 REVERSE |
| **AT4G24250.1** | **MLO13** | | 478 | N | 7 | OUT | 7 | OUT | N | \| Symbols: ATMLO13, MLO13 \| MLO13 (MILDEW RESISTANCE LOCUS O 13); calmodulin binding \| chr4:12575018-12577527 REVERSE |
| **AT4G25010.1** | | | 281 | N | 7 | OUT | 7 | OUT | N | \| Symbols:  \| nodulin MtN3 family protein \| chr4:12854640-12856361 REVERSE |
| **AT4G25750.1** | | | 577 | N | 7 | OUT | 8 | IN | N | \| Symbols:  \| ABC transporter family protein \| chr4:13110636-13112369 REVERSE |
| **AT4G26770.1** | | | 471 | N | 8 | IN | 8 | OUT | N | \| Symbols:  \| phosphatidate cytidylyltransferase, putative / CDP-diglyceride synthetase, putative \| chr4:13482586-13484703 FORWARD |
| **AT4G28370.1** | | | 562 | N | 8 | IN | 7 | IN | N | \| Symbols:  \| zinc ion binding \| chr4:14035022-14039128 FORWARD |
| **AT4G30850.1** | **HHP2** | **HHP2** | 358 | N | 8 | OUT | 7 | IN | N | \| Symbols: HHP2 \| HHP2 (HEPTAHELICAL TRANSMEMBRANE PROTEIN2) \| chr4:15020546-15022284 REVERSE |
| *AT4G30850.2* | *HHP2* | | *358* | *N* | *8* | *OUT* | *7* | *IN* | *N* | *\| Symbols: HHP2 \| HHP2 (HEPTAHELICAL TRANSMEMBRANE PROTEIN2) \| chr4:15020546-15022284 REVERSE* |
| **AT4G32650.2** | | | 597 | N | 7 | IN | 7 | IN | N | \| Symbols: KAT3, ATKC1 \| ATKC1 (ARABIDOPSIS THALIANA K+ RECTIFYING CHANNEL 1); cyclic nucleotide binding / inward rectifier potassium channel \| chr4:15751533-15754802 REVERSE |
| **AT4G36830.1** | | | 289 | N | 7 | OUT | 7 | OUT | N | \| Symbols:  \| GNS1/SUR4 membrane family protein \| chr4:17349515-17350384 FORWARD |
| **AT4G36850.1** | | | 374 | N | 7 | OUT | 7 | OUT | N | \| Symbols:  \| PQ-loop repeat family protein / transmembrane family protein \| chr4:17353418-17355824 REVERSE |
| **AT4G37680.1** | **HHP4** | | 385 | N | 7 | IN | 7 | IN | N | \| Symbols: HHP4 \| HHP4 (heptahelical protein 4); receptor \| chr4:17701225-17702562 FORWARD |

| | | | | | | | | | | |
|---|---|---|---|---|---|---|---|---|---|---|
| **AT5G01240.2** | | | 408 | N | 10 | OUT | 10 | OUT | G | \| Symbols:  \| amino acid permease, putative \| chr5:98662-101492 FORWARD |
| **AT5G02630.1** | | **Cand6** | 428 | Y | 7 | OUT | 8 | OUT | G | \| Symbols:  \| similar to unknown protein [Arabidopsis thaliana] (TAIR:AT5G18520.1); similar to unknown protein [Arabidopsis thaliana] (TAIR:AT3G09570.1); similar to unnamed protein product [Vitis vinifera] (GB:CAO18079.1); contains InterPro domain Transmembrane receptor, eukaryota; (InterPro:IPR009637) \| chr5:591796-593082 FORWARD |
| **AT5G05350.1** | | | 526 | N | 7 | OUT | 6 | IN | G | \| Symbols:  \| similar to unknown protein [Arabidopsis thaliana] (TAIR:AT3G10980.1); similar to unnamed protein product [Vitis vinifera] (GB:CAO41459.1); contains InterPro domain Protein of unknown function Cys-rich (InterPro:IPR006461) \| chr5:1585758-1587338 FORWARD |
| **AT5G07250.1** | | | 346 | N | 7 | IN | 7 | IN | N | \| Symbols:  \| rhomboid family protein \| chr5:2274438-2275936 REVERSE |
| **AT5G07630.1** | | | 401 | N | 8 | IN | 8 | IN | N | \| Symbols:  \| nuclear division RFT family protein \| chr5:2412033-2414524 FORWARD |
| **AT5G08090.1** | | | 322 | N | 8 | IN | 7 | IN | N | \| Symbols:  \| unknown protein \| chr5:2591712-2592900 FORWARD |
| **AT5G11870.1** | | | 262 | N | 7 | OUT | 8 | IN | N | \| Symbols:  \| similar to SAG18 (SENESCENCE ASSOCIATED GENE 18) [Arabidopsis thaliana] (TAIR:AT1G71190.1); similar to unnamed protein product [Vitis vinifera] (GB:CAO43822.1) \| chr5:3825721-3827242 FORWARD |
| **AT5G11960.1** | | | 344 | N | 8 | IN | 8 | IN | N | \| Symbols:  \| similar to hypothetical protein [Vitis vinifera] (GB:CAN81798.1); contains InterPro domain Protein of unknown function DUF6, transmembrane; (InterPro:IPR000620) \| chr5:3858853-3861463 REVERSE |
| **AT5G13170.1** | | | 292 | N | 7 | OUT | 7 | OUT | N | \| Symbols:  \| nodulin MtN3 family protein \| chr5:4181334-4183174 REVERSE |
| **AT5G15240.2** | | | 313 | N | 7 | IN | 8 | OUT | N | \| Symbols:  \| amino acid transporter family protein \| chr5:4947765-4950214 FORWARD |
| **AT5G18520.1** | | **Cand7** | 440 | Y | 7 | OUT | 8 | IN | G | \| Symbols:  \| similar to unknown protein [Arabidopsis thaliana] (TAIR:AT3G09570.1); similar to unnamed protein product [Vitis vinifera] (GB:CAO18079.1); contains InterPro domain Transmembrane receptor, eukaryota; (InterPro:IPR009637) \| chr5:6145029-6146351 FORWARD |
| **AT5G19870.1** | | | 276 | N | 7 | OUT | 7 | OUT | N | \| Symbols:  \| similar to unknown protein [Arabidopsis thaliana] (TAIR:AT1G55230.1); similar to unnamed protein product [Vitis vinifera] (GB:CAO63741.1); contains InterPro domain Protein of unknown function DUF716 (InterPro:IPR006904) \| chr5:6716184-6717014 REVERSE |
| **AT5G20270.1** | **HHP1** | | 332 | N | 7 | IN | 7 | IN | N | \| Symbols: HHP1 \| HHP1 (HEPTAHELICAL TRANSMEMBRANE PROTEIN1) \| chr5:6841027-6842658 REVERSE |
| **AT5G23660.1** | | | 285 | N | 7 | OUT | 7 | OUT | N | \| Symbols: MTN3 \| MTN3 (ARABIDOPSIS HOMOLOG OF MEDICAGO TRUNCATULA MTN3) \| chr5:7971939-7973799 REVERSE |
| **AT5G23990.1** | | | 657 | N | 8 | IN | 7 | OUT | N | \| Symbols: ATFRO5, FRO5 \| ATFRO5/FRO5 (FERRIC REDUCTION OXIDASE 5); ferric-chelate reductase \| chr5:8105568-8108593 REVERSE |
| **AT5G26740.1** | | **Cand9** | 422 | N | 7 | OUT | 7 | OUT | N | \| Symbols:  \| similar to unknown protein [Arabidopsis thaliana] (TAIR:AT3G05940.1); similar to unknown [Populus trichocarpa] (GB:ABK95399.1); contains InterPro domain Protein of unknown function DUF300 (InterPro:IPR005178) \| chr5:9292439-9294410 FORWARD |
| *AT5G26740.2* | | | *422* | *N* | *7* | *OUT* | *7* | *OUT* | *N* | *\| Symbols:  \| similar to unknown protein [Arabidopsis thaliana] (TAIR:AT3G05940.1); similar to unknown [Populus trichocarpa] (GB:ABK95399.1); contains InterPro domain Protein of unknown function DUF300 (InterPro:IPR005178) \| chr5:9292439-9294410 FORWARD* |
| *AT5G26740.3* | | | *422* | *N* | *7* | *OUT* | *7* | *OUT* | *N* | *\| Symbols:  \| similar to unknown protein [Arabidopsis thaliana] (TAIR:AT3G05940.1); similar to unknown [Populus trichocarpa] (GB:ABK95399.1); contains InterPro domain Protein of unknown function DUF300 (InterPro:IPR005178) \| chr5:9292439-9294410 FORWARD* |
| **AT5G27210.1** | | **Cand8** | 297 | N | 7 | OUT | 7 | OUT | G | \| Symbols:  \| similar to transmembrane protein, putative [Arabidopsis thaliana] (TAIR:AT3G05010.1); similar to unnamed protein product [Vitis vinifera] (GB:CAO15118.1) \| chr5:9572336-9574078 REVERSE |
| **AT5G33320.1** | | | 408 | N | 8 | IN | 7 | IN | N | \| Symbols: PPT, ARAPPT, CUE1 \| CUE1 (CAB UNDEREXPRESSED 1); antiporter/ triose-phosphate transmembrane transporter \| chr5:12606180-12608638 FORWARD |
| **AT5G35460.1** | | | 381 | N | 8 | IN | 8 | IN | N | \| Symbols:  \| similar to unknown [Populus trichocarpa] (GB:ABK95300.1); contains domain Acetyl-CoA synthetase-like (SSF56801) \| chr5:13689828-13691776 FORWARD |
| **AT5G37310.1** | | | 593 | Y | 9 | OUT | 9 | OUT | G | \| Symbols:  \| transporter \| chr5:14790066-14793323 REVERSE |
| **AT5G42090.1** | | | 439 | Y | 7 | OUT | 7 | OUT | G | \| Symbols:  \| similar to unknown protein [Arabidopsis thaliana] (TAIR:AT3G09570.1); similar to unnamed protein product [Vitis vinifera] (GB:CAO62906.1); contains InterPro domain Transmembrane receptor, eukaryota; (InterPro:IPR009637) \| chr5:16844058-16845377 FORWARD |
| **AT5G45105.1** | | | 315 | Y | 7 | OUT | 8 | IN | N | \| Symbols: ZIP8 \| ZIP8 (ZINC TRANSPORTER 8 PRECURSOR); cation transmembrane transporter \| chr5:18240680-18242173 REVERSE |
| **AT5G47900.2** | | | 359 | N | 7 | IN | 7 | IN | N | \| Symbols:  \| similar to unknown protein [Arabidopsis thaliana] (TAIR:AT5G27730.1); similar to unnamed protein product [Vitis vinifera] (GB:CAO68737.1) \| chr5:19409634-19411387 FORWARD |
| **AT5G50375.1** | | | 280 | N | 7 | IN | 7 | IN | N | \| Symbols: CPI1 \| CPI1 (CYCLOPROPYL ISOMERASE) \| chr5:20529082-20531157 FORWARD |
| **AT5G50790.1** | | | 289 | N | 7 | OUT | 7 | OUT | N | \| Symbols:  \| nodulin MtN3 family protein \| chr5:20673687-20675053 REVERSE |
| **AT5G50800.1** | | | 294 | N | 7 | OUT | 7 | OUT | N | \| Symbols:  \| nodulin MtN3 family protein \| chr5:20682506-20684366 REVERSE |
| **AT5G53190.1** | | | 263 | N | 7 | OUT | 7 | OUT | N | \| Symbols:  \| nodulin MtN3 family protein \| chr5:21589643-21591510 REVERSE |
| **AT5G53760.1** | **MLO11** | | 573 | N | 7 | OUT | 7 | OUT | N | \| Symbols: ATMLO11, MLO11 \| MLO11 (MILDEW RESISTANCE LOCUS O 11); calmodulin binding \| chr5:21840281-21843515 FORWARD |
| *AT5G53760.2* | *MLO11* | | *573* | *N* | *7* | *OUT* | *7* | *OUT* | *N* | *\| Symbols: ATMLO11, MLO11 \| MLO11 (MILDEW RESISTANCE LOCUS O 11); calmodulin binding \| chr5:21840281-21843515 FORWARD* |
| **AT5G55370.1** | | | 343 | N | 8 | IN | 8 | OUT | N | \| Symbols:  \| long-chain-alcohol O-fatty-acyltransferase family protein / wax synthase family protein \| chr5:22462311-22463342 REVERSE |
| **AT5G57100.1** | | | 390 | N | 10 | IN | 10 | IN | G | \| Symbols:  \| transporter-related \| chr5:23123789-23125666 REVERSE |
| **AT5G59500.1** | | | 396 | N | 8 | IN | 7 | IN | N | \| Symbols:  \| protein-S-isoprenylcysteine O-methyltransferase \| chr5:24003250-24004440 FORWARD |
| **AT5G62850.1** | | | 240 | N | 7 | OUT | 7 | OUT | N | \| Symbols: ATVEX1 \| ATVEX1 (VEGETATIVE CELL EXPRESSED1) \| chr5:25247430-25248753 REVERSE |
| **AT5G62960.1** | | | 347 | N | 7 | OUT | 7 | OUT | N | \| Symbols:  \| similar to unknown protein [Arabidopsis thaliana] (TAIR:AT1G10660.3); similar to unknown protein [Arabidopsis thaliana] (TAIR:AT1G10660.1); similar to unknown protein [Arabidopsis thaliana] (TAIR:AT1G10660.4); similar to unnamed protein product [Vitis vinifera] (GB:CAO64308.1) \| chr5:25286670-25288759 FORWARD |
| **AT5G65000.2** | | | 260 | Y | 7 | OUT | 8 | IN | N | \| Symbols:  \| nucleotide-sugar transporter family protein \| chr5:25982722-25984533 REVERSE |
| **AT5G65970.1** | **MLO10** | | 569 | Y | 7 | OUT | 9 | OUT | R | \| Symbols: ATMLO10, MLO10 \| MLO10 (MILDEW RESISTANCE LOCUS O 10); calmodulin binding \| chr5:26404735-26407633 REVERSE |
| **ATCG01040.1** | | | 328 | N | 8 | OUT | 8 | OUT | N | \| Symbols: YCF5 \| hypothetical protein \| chrC:114461-115447 FORWARD |
