# Supplementary material for: 7TMRmine: a Web server for hierarchical mining of 7TMR proteins
Source: BMC Genomics. 2009 Jun 19;10:275. doi: 10.1186/1471-2164-10-275 (PMC2718930; doi:10.1186/1471-2164-10-275)
Supplement: Additional file 4 — 7TMR candidate proteins identified from the Oryza sativa genome. 84 proteins were obtained by combining the results of eight classifiers and two TM-prediction methods. [file 1471-2164-10-275-S4.pdf]

Table S3. 7TMR candidate proteins identified from the *Oryza sativa* genome. 84 proteins were obtained by combining the results of eight classifiers and two TM prediction methods. 64 proteins (listed with yellow background) were identified by taking the intersection of positives predicted by "6 classifiers" and "7-8 TM". Another 20 proteins (listed with white background) were identified taking the intersection of positives predicted by "SAM2+GPCRHMM" and "5-10 TM".

"Gookin": Names of the 8 high-ranking GPCR proteins identified by Gookin et al. (2008).
"Phobius_SIG": Signal peptide prediction by Phobius (Y: yes, N: no).
"Phobius": Number of transmembrane regions predicted by Phobius.
"Phobius_Nterm": Location of N-terminus predicted by Phobius (IN: intracellular, OUT: extracellular).
"HMMTOP": Number of transmembrane regions predicted by HMMTOP.
"HMMTOP_Nterm": Location of N-terminus predicted by HMMTOP (IN: intracellular, OUT: extracellular).
"GPCRHMM": GPCR prediction by GPCRHMM (G: GPCR, N: non-GPCR).

SeqID	Gookin	Length (aa)	Phobius _SIG	Phobius	Phobius _Nterm	HMMTOP	HMMTOP _Nterm	GPCRHMM	Description
**gi|115434780|ref|NP_001042148.1|**		385	N	8	IN	7	IN	R	Os01g0171800 [Oryza sativa (japonica cultivar-group)]
**gi|115434858|ref|NP_001042187.1|**		371	N	7	OUT	7	OUT	R	Os01g0177900 [Oryza sativa (japonica cultivar-group)]
**gi|115437336|ref|NP_001043270.1|**		243	Y	7	OUT	7	OUT	R	Os01g0541800 [Oryza sativa (japonica cultivar-group)]
**gi|115437960|ref|NP_001043423.1|**		371	N	8	IN	8	IN	R	Os01g0585100 [Oryza sativa (japonica cultivar-group)]
**gi|115438366|ref|NP_001043522.1|**		254	N	7	OUT	7	OUT	R	Os01g0605700 [Oryza sativa (japonica cultivar-group)]
**gi|115439407|ref|NP_001043983.1|**		230	N	7	OUT	7	OUT	R	Os01g0700100 [Oryza sativa (japonica cultivar-group)]
**gi|115439957|ref|NP_001044258.1|**	**Os01g54784.1**	361	N	7	OUT	7	OUT	G	Os01g0751300 [Oryza sativa (japonica cultivar-group)]
**gi|115440045|ref|NP_001044302.1|**		425	N	7	OUT	8	OUT	R	Os01g0758400 [Oryza sativa (japonica cultivar-group)]
**gi|115440907|ref|NP_001044733.1|**	**Os01g61970.1**	449	Y	7	OUT	9	OUT	G	Os01g0836800 [Oryza sativa (japonica cultivar-group)]
**gi|115441485|ref|NP_001045022.1|**	**Os01g66190.1**	397	N	7	OUT	7	OUT	G	Os01g0885200 [Oryza sativa (japonica cultivar-group)]
**gi|115441521|ref|NP_001045040.1|**		502	N	7	OUT	7	OUT	R	Os01g0888600 [Oryza sativa (japonica cultivar-group)]
**gi|115442393|ref|NP_001045476.1|**		294	Y	7	OUT	7	IN	R	Os01g0962200 [Oryza sativa (japonica cultivar-group)]
**gi|115443813|ref|NP_001045686.1|**		343	N	7	OUT	8	IN	R	Os02g0117400 [Oryza sativa (japonica cultivar-group)]
**gi|115444011|ref|NP_001045785.1|**		362	Y	7	OUT	7	OUT	R	Os02g0130600 [Oryza sativa (japonica cultivar-group)]
**gi|115444835|ref|NP_001046197.1|**		386	N	7	OUT	7	OUT	R	Os02g0197200 [Oryza sativa (japonica cultivar-group)]
**gi|115445495|ref|NP_001046527.1|**		249	N	7	OUT	7	OUT	R	Os02g0272600 [Oryza sativa (japonica cultivar-group)]
**gi|115446237|ref|NP_001046898.1|**		268	N	7	OUT	7	OUT	R	Os02g0498300 [Oryza sativa (japonica cultivar-group)]
**gi|115446649|ref|NP_001047104.1|**		590	Y	9	OUT	10	IN	G	Os02g0552000 [Oryza sativa (japonica cultivar-group)]
**gi|115446783|ref|NP_001047171.1|**		449	N	7	IN	8	IN	R	Os02g0566400 [Oryza sativa (japonica cultivar-group)]
**gi|115446853|ref|NP_001047206.1|**		393	N	8	IN	7	OUT	R	Os02g0574500 [Oryza sativa (japonica cultivar-group)]
**gi|115447373|ref|NP_001047466.1|**		296	N	7	OUT	8	IN	R	Os02g0622200 [Oryza sativa (japonica cultivar-group)]
**gi|115449063|ref|NP_001048311.1|**		428	N	7	OUT	7	OUT	R	Os02g0781600 [Oryza sativa (japonica cultivar-group)]
**gi|115449169|ref|NP_001048364.1|**		414	Y	9	OUT	10	IN	G	Os02g0792900 [Oryza sativa (japonica cultivar-group)]
**gi|115450317|ref|NP_001048759.1|**		325	N	7	IN	7	IN	R	Os03g0116400 [Oryza sativa (japonica cultivar-group)]
**gi|115450373|ref|NP_001048787.1|**		281	N	7	OUT	7	OUT	R	Os03g0120300 [Oryza sativa (japonica cultivar-group)]
**gi|115450489|ref|NP_001048845.1|**		555	N	7	OUT	9	OUT	R	Os03g0129100 [Oryza sativa (japonica cultivar-group)]
**gi|115451741|ref|NP_001049471.1|**		403	N	7	IN	7	IN	R	Os03g0232900 [Oryza sativa (japonica cultivar-group)]
**gi|115451783|ref|NP_001049492.1|**		595	Y	9	OUT	10	IN	G	Os03g0237000 [Oryza sativa (japonica cultivar-group)]
**gi|115452861|ref|NP_001050031.1|**		494	Y	7	OUT	8	IN	R	Os03g0334800 [Oryza sativa (japonica cultivar-group)]
**gi|115454871|ref|NP_001051036.1|**		291	N	7	OUT	8	OUT	R	Os03g0708400 [Oryza sativa (japonica cultivar-group)]
**gi|115455557|ref|NP_001051379.1|**		262	N	7	OUT	7	OUT	R	Os03g0766000 [Oryza sativa (japonica cultivar-group)]
**gi|115455961|ref|NP_001051581.1|**		393	N	8	IN	8	IN	R	Os03g0800000 [Oryza sativa (japonica cultivar-group)]
**gi|115458580|ref|NP_001052890.1|**	**Os04g36630.1**	421	Y	7	OUT	8	IN	G	Os04g0443700 [Oryza sativa (japonica cultivar-group)]
**gi|115458586|ref|NP_001052893.1|**		476	N	7	OUT	7	OUT	R	Os04g0444400 [Oryza sativa (japonica cultivar-group)]
**gi|115458802|ref|NP_001053001.1|**		340	Y	7	OUT	7	IN	R	Os04g0462300 [Oryza sativa (japonica cultivar-group)]

**gi|115459130|ref|NP_001053165.1|**		293	N	7	IN	7	IN	R	Os04g0490600 [Oryza sativa (japonica cultivar-group)]
**gi|115460324|ref|NP_001053762.1|**		404	N	7	OUT	7	OUT	R	Os04g0600800 [Oryza sativa (japonica cultivar-group)]
**gi|115462691|ref|NP_001054945.1|**		349	Y	7	OUT	8	IN	R	Os05g0220100 [Oryza sativa (japonica cultivar-group)]
**gi|115463787|ref|NP_001055493.1|**		264	N	7	IN	7	IN	R	Os05g0402300 [Oryza sativa (japonica cultivar-group)]
**gi|115464335|ref|NP_001055767.1|**		458	Y	7	OUT	8	IN	G	Os05g0462500 [Oryza sativa (japonica cultivar-group)]
**gi|115464347|ref|NP_001055773.1|**		275	N	8	IN	8	OUT	G	Os05g0463700 [Oryza sativa (japonica cultivar-group)]
**gi|115464459|ref|NP_001055829.1|**	**Os05g39730.1**	554	N	7	OUT	7	OUT	G	Os05g0474900 [Oryza sativa (japonica cultivar-group)]
**gi|115465994|ref|NP_001056596.1|**		388	N	8	OUT	7	IN	R	Os06g0112800 [Oryza sativa (japonica cultivar-group)]
**gi|115466198|ref|NP_001056698.1|**	**Os06g04130.1**	454	Y	7	OUT	9	OUT	G	Os06g0132100 [Oryza sativa (japonica cultivar-group)]
**gi|115466482|ref|NP_001056840.1|**		281	N	7	OUT	7	OUT	R	Os06g0153200 [Oryza sativa (japonica cultivar-group)]
**gi|115466940|ref|NP_001057069.1|**	**Os06g09930.1**	321	Y	7	OUT	7	OUT	G	Os06g0199800 [Oryza sativa (japonica cultivar-group)]
**gi|115466978|ref|NP_001057088.1|**		305	N	6	OUT	6	IN	G	Os06g0204400 [Oryza sativa (japonica cultivar-group)]
**gi|115468130|ref|NP_001057664.1|**		528	N	7	OUT	9	OUT	R	Os06g0486300 [Oryza sativa (japonica cultivar-group)]
**gi|115468562|ref|NP_001057880.1|**		416	N	8	IN	8	IN	R	Os06g0563900 [Oryza sativa (japonica cultivar-group)]
**gi|115468604|ref|NP_001057901.1|**		380	N	9	OUT	9	OUT	G	Os06g0568000 [Oryza sativa (japonica cultivar-group)]
**gi|115469082|ref|NP_001058140.1|**		452	N	7	IN	7	IN	R	Os06g0635200 [Oryza sativa (japonica cultivar-group)]
**gi|115469246|ref|NP_001058222.1|**		645	Y	9	OUT	10	IN	G	Os06g0650600 [Oryza sativa (japonica cultivar-group)]
**gi|115470169|ref|NP_001058683.1|**	**Os07g01250.1**	286	N	7	OUT	7	OUT	R	Os07g0102400 [Oryza sativa (japonica cultivar-group)]
**gi|115470855|ref|NP_001059026.1|**		244	N	7	IN	7	IN	R	Os07g0177300 [Oryza sativa (japonica cultivar-group)]
**gi|115471777|ref|NP_001059487.1|**		1093	N	8	OUT	8	OUT	R	Os07g0424400 [Oryza sativa (japonica cultivar-group)]
**gi|115472177|ref|NP_001059687.1|**		380	N	7	OUT	7	OUT	R	Os07g0493400 [Oryza sativa (japonica cultivar-group)]
**gi|115472297|ref|NP_001059747.1|**		434	N	7	OUT	8	OUT	R	Os07g0509200 [Oryza sativa (japonica cultivar-group)]
**gi|115472429|ref|NP_001059813.1|**		497	N	7	OUT	7	OUT	R	Os07g0522500 [Oryza sativa (japonica cultivar-group)]
**gi|115473837|ref|NP_001060517.1|**		393	N	7	OUT	7	OUT	R	Os07g0658100 [Oryza sativa (japonica cultivar-group)]
**gi|115474401|ref|NP_001060797.1|**		350	N	7	OUT	8	IN	R	Os08g0107400 [Oryza sativa (japonica cultivar-group)]
**gi|115474625|ref|NP_001060909.1|**		483	Y	7	OUT	8	IN	R	Os08g0128200 [Oryza sativa (japonica cultivar-group)]
**gi|115474915|ref|NP_001061054.1|**		314	N	8	IN	8	IN	G	Os08g0162000 [Oryza sativa (japonica cultivar-group)]
**gi|115477619|ref|NP_001062405.1|**		475	N	7	OUT	7	OUT	R	Os08g0544400 [Oryza sativa (japonica cultivar-group)]
**gi|115477743|ref|NP_001062467.1|**		646	Y	9	OUT	10	IN	G	Os08g0554900 [Oryza sativa (japonica cultivar-group)]
**gi|115477747|ref|NP_001062469.1|**		590	Y	9	OUT	10	IN	G	Os08g0555200 [Oryza sativa (japonica cultivar-group)]
**gi|115479367|ref|NP_001063277.1|**		532	Y	7	OUT	8	IN	R	Os09g0439700 [Oryza sativa (japonica cultivar-group)]
**gi|115480625|ref|NP_001063906.1|**		646	Y	9	OUT	10	IN	G	Os09g0557800 [Oryza sativa (japonica cultivar-group)]
**gi|115480711|ref|NP_001063949.1|**		332	Y	7	OUT	7	IN	R	Os09g0565600 [Oryza sativa (japonica cultivar-group)]
**gi|115480894|ref|NP_001064040.1|**		585	Y	9	OUT	9	OUT	G	Os10g0112600 [Oryza sativa (japonica cultivar-group)]
**gi|115481414|ref|NP_001064300.1|**		316	N	7	OUT	7	OUT	R	Os10g0200700 [Oryza sativa (japonica cultivar-group)]
**gi|115482244|ref|NP_001064715.1|**		859	Y	7	OUT	7	IN	R	Os10g0446200 [Oryza sativa (japonica cultivar-group)]
**gi|115483024|ref|NP_001065105.1|**		347	Y	7	OUT	8	IN	R	Os10g0524100 [Oryza sativa (japonica cultivar-group)]
**gi|115483162|ref|NP_001065174.1|**		294	Y	7	OUT	8	IN	R	Os10g0537500 [Oryza sativa (japonica cultivar-group)]
**gi|115483192|ref|NP_001065189.1|**		537	N	5	OUT	6	IN	R	Os10g0541000 [Oryza sativa (japonica cultivar-group)]
**gi|115483817|ref|NP_001065570.1|**		322	N	7	IN	8	IN	R	Os11g0112900 [Oryza sativa (japonica cultivar-group)]
**gi|115484053|ref|NP_001065688.1|**		278	N	7	OUT	7	IN	R	Os11g0136300 [Oryza sativa (japonica cultivar-group)]
**gi|115484301|ref|NP_001065812.1|**		358	N	8	IN	7	OUT	R	Os11g0159500 [Oryza sativa (japonica cultivar-group)]
**gi|115484333|ref|NP_001065828.1|**		529	N	8	IN	7	IN	R	Os11g0162300 [Oryza sativa (japonica cultivar-group)]
**gi|115484335|ref|NP_001065829.1|**		337	N	8	IN	8	IN	R	Os11g0162900 [Oryza sativa (japonica cultivar-group)]
**gi|115484471|ref|NP_001065897.1|**		593	Y	9	OUT	10	IN	G	Os11g0181100 [Oryza sativa (japonica cultivar-group)]
**gi|115486807|ref|NP_001068547.1|**		374	N	7	IN	7	IN	R	Os11g0704800 [Oryza sativa (japonica cultivar-group)]
**gi|115487610|ref|NP_001066292.1|**		598	Y	9	OUT	9	OUT	G	Os12g0175700 [Oryza sativa (japonica cultivar-group)]
**gi|115488580|ref|NP_001066777.1|**		458	N	8	IN	8	IN	R	Os12g0484600 [Oryza sativa (japonica cultivar-group)]
**gi|115488760|ref|NP_001066867.1|**		418	N	7	OUT	7	OUT	R	Os12g0511200 [Oryza sativa (japonica cultivar-group)]
