# Supplementary material for: 7TMRmine: a Web server for hierarchical mining of 7TMR proteins
Source: BMC Genomics. 2009 Jun 19;10:275. doi: 10.1186/1471-2164-10-275 (PMC2718930; doi:10.1186/1471-2164-10-275)
Supplement: Additional file 5 — 7TMR candidate proteins identified from the Populus trichocarpa genome. 153 proteins were obtained by combining the results of eight classifiers and two TM-prediction methods. [file 1471-2164-10-275-S5.pdf]

Table S4. 7TMR candidate proteins identified from the *Populus trichocarpa* genome. 153 proteins were obtained by combining the results of eight classifiers and two TM prediction methods. 125 proteins (listed with yellow background) were identified by taking the intersection of positives predicted by "6 classifiers" and "7-8 TM". Another 28 proteins (listed with white background) were identified taking the intersection of positives predicted by "SAM2+GPCRHMM" and "5-10 TM".

"Gookin": Names of the 17 high-ranking GPCR proteins identified by Gookin et al. (2008).
"Phobius_SIG": Signal peptide prediction by Phobius (Y: yes, N: no).
"Phobius": Number of transmembrane regions predicted by Phobius.
"Phobius_Nterm": Location of N-terminus predicted by Phobius (IN: intracellular, OUT: extracellular).
"HMMTOP": Number of transmembrane regions predicted by HMMTOP.
"HMMTOP_Nterm": Location of N-terminus predicted by HMMTOP (IN: intracellular, OUT: extracellular).
"GPCRHMM": GPCR prediction by GPCRHMM (G: GPCR, N: non-GPCR).

SeqID	Gookin	Length (aa)	Phobius_ SIG	Phobius	Phobius_ Nterm	HMMTOP	HMMTOP_ Nterm	GPCRHMM
jgi|Poptr1_1|172604|gw1.I.1204.1		324	N	8	IN	7	IN	R
jgi|Poptr1_1|173974|gw1.I.2574.1		709	N	7	OUT	8	IN	R
jgi|Poptr1_1|177980|gw1.I.6580.1		282	N	7	OUT	7	OUT	R
jgi|Poptr1_1|177987|gw1.I.6587.1		271	N	7	OUT	7	OUT	R
jgi|Poptr1_1|179233|gw1.I.7833.1		217	N	7	OUT	7	OUT	R
jgi|Poptr1_1|197868|gw1.IV.2957.1		434	N	7	OUT	8	OUT	R
jgi|Poptr1_1|202620|gw1.IX.3085.1		566	N	7	OUT	8	IN	R
jgi|Poptr1_1|205267|gw1.V.668.1	205267	344	N	7	OUT	7	OUT	G
jgi|Poptr1_1|206779|gw1.V.2180.1		500	N	7	OUT	9	OUT	R
jgi|Poptr1_1|206792|gw1.V.2193.1		437	N	7	OUT	8	IN	R
jgi|Poptr1_1|208168|gw1.V.3569.1		519	N	7	OUT	9	OUT	R
jgi|Poptr1_1|215806|gw1.VII.111.1		378	N	7	IN	8	OUT	R
jgi|Poptr1_1|216224|gw1.VII.529.1		516	N	8	OUT	7	IN	R
jgi|Poptr1_1|216767|gw1.VII.1072.1		414	N	8	IN	7	IN	R
jgi|Poptr1_1|218443|gw1.VII.2748.1		531	N	7	OUT	9	OUT	R
jgi|Poptr1_1|219003|gw1.VII.3308.1		371	N	7	OUT	7	OUT	R
jgi|Poptr1_1|225580|gw1.X.277.1		248	N	7	IN	7	IN	R
jgi|Poptr1_1|226271|gw1.X.968.1		327	N	5	OUT	6	IN	G
jgi|Poptr1_1|227902|gw1.X.2599.1		486	N	7	OUT	9	OUT	R
jgi|Poptr1_1|230624|gw1.X.5321.1		332	N	7	OUT	7	OUT	R
jgi|Poptr1_1|233369|gw1.XI.1349.1		262	N	8	IN	7	OUT	R
jgi|Poptr1_1|235962|gw1.XI.3942.1		556	N	7	OUT	7	OUT	R
jgi|Poptr1_1|241510|gw1.XIII.1759.1	241510	436	Y	7	OUT	7	OUT	G
jgi|Poptr1_1|242087|gw1.XIII.2336.1		476	N	7	OUT	9	OUT	R
jgi|Poptr1_1|251548|gw1.XV.984.1		283	N	7	OUT	8	OUT	R
jgi|Poptr1_1|252572|gw1.XV.2008.1		404	N	7	IN	8	IN	R
jgi|Poptr1_1|252617|gw1.XV.2053.1		277	N	7	IN	7	IN	R
jgi|Poptr1_1|252864|gw1.XV.2300.1		205	N	7	OUT	7	OUT	R

**jgi|Poptr1_1|254201|gw1.XVI.140.1**		468	N	7	OUT	7	IN	R
**jgi|Poptr1_1|254437|gw1.XVI.376.1**	**254437**	455	Y	7	OUT	7	OUT	G
**jgi|Poptr1_1|256636|gw1.XVI.2575.1**	**256636**	424	N	7	OUT	7	OUT	G
**jgi|Poptr1_1|256812|gw1.XVI.2751.1**		474	N	7	OUT	9	OUT	R
**jgi|Poptr1_1|258330|gw1.XVII.321.1**		276	N	8	OUT	8	IN	R
**jgi|Poptr1_1|259524|gw1.XVIII.65.1**		304	N	7	IN	7	IN	R
**jgi|Poptr1_1|262559|gw1.XVIII.3100.1**		334	N	7	IN	7	IN	R
**jgi|Poptr1_1|263939|gw1.11155.4.1**		362	Y	7	OUT	7	OUT	R
**jgi|Poptr1_1|265277|gw1.12017.3.1**		268	N	7	OUT	7	OUT	R
**jgi|Poptr1_1|265459|gw1.121.129.1**		330	N	8	IN	8	OUT	R
**jgi|Poptr1_1|267303|gw1.12729.1.1**		259	N	7	OUT	7	OUT	R
**jgi|Poptr1_1|267622|gw1.12918.1.1**		259	N	7	OUT	7	OUT	R
**jgi|Poptr1_1|268014|gw1.131.16.1**		419	N	7	OUT	7	IN	R
**jgi|Poptr1_1|269409|gw1.13532.4.1**		316	N	8	OUT	8	OUT	R
**jgi|Poptr1_1|269792|gw1.13731.1.1**		259	N	7	OUT	7	OUT	R
**jgi|Poptr1_1|269877|gw1.13864.5.1**		318	N	8	OUT	8	OUT	R
**jgi|Poptr1_1|271644|gw1.147.212.1**		409	N	7	IN	7	IN	R
**jgi|Poptr1_1|272233|gw1.150.48.1**		433	N	7	OUT	8	OUT	R
**jgi|Poptr1_1|272274|gw1.150.89.1**	**272274**	397	N	7	OUT	8	IN	G
**jgi|Poptr1_1|272750|gw1.152.219.1**		284	N	7	IN	7	OUT	R
**jgi|Poptr1_1|275359|gw1.1708.2.1**		288	N	8	IN	8	IN	R
**jgi|Poptr1_1|276782|gw1.187.17.1**		430	N	7	OUT	8	OUT	R
**jgi|Poptr1_1|277455|gw1.197.4.1**		217	N	7	OUT	7	OUT	R
**jgi|Poptr1_1|279432|gw1.2197.2.1**	**279432**	358	N	7	OUT	7	OUT	R
**jgi|Poptr1_1|286694|gw1.40.454.1**		277	N	7	OUT	7	OUT	R
**jgi|Poptr1_1|288106|gw1.41.591.1**		351	N	7	OUT	7	OUT	R
**jgi|Poptr1_1|289338|gw1.44.551.1**		588	Y	9	OUT	9	OUT	G
**jgi|Poptr1_1|290602|gw1.5147.2.1**		304	N	8	IN	8	OUT	R
**jgi|Poptr1_1|294952|gw1.70.405.1**	**294952**	281	N	7	OUT	7	OUT	R
**jgi|Poptr1_1|408817|gw1.II.152.1**		501	N	7	OUT	7	OUT	R
**jgi|Poptr1_1|412008|gw1.II.3343.1**		453	N	7	OUT	8	IN	R
**jgi|Poptr1_1|414129|gw1.III.1232.1**		598	Y	9	OUT	10	IN	G
**jgi|Poptr1_1|414177|gw1.III.1280.1**		254	N	7	OUT	7	OUT	R
**jgi|Poptr1_1|414178|gw1.III.1281.1**		264	N	7	OUT	7	OUT	R
**jgi|Poptr1_1|414904|gw1.III.2007.1**		223	N	7	OUT	7	OUT	R
**jgi|Poptr1_1|417284|gw1.VI.1657.1**		358	N	7	OUT	7	OUT	R
**jgi|Poptr1_1|417676|gw1.VI.2049.1**		386	Y	8	OUT	8	OUT	R
**jgi|Poptr1_1|419541|gw1.VIII.969.1**		533	N	7	OUT	8	OUT	R
**jgi|Poptr1_1|421484|gw1.VIII.2912.1**		553	N	9	OUT	9	OUT	G
**jgi|Poptr1_1|421765|gw1.XII.225.1**		220	N	7	OUT	7	OUT	R
**jgi|Poptr1_1|423394|gw1.XII.1854.1**		321	N	7	OUT	7	OUT	R
**jgi|Poptr1_1|551452|eugene3.00020767**		282	N	8	IN	7	OUT	R
**jgi|Poptr1_1|552585|eugene3.00021900**		320	N	7	OUT	7	OUT	R

**jgi|Poptr1_1|554569|eugene3.00031202**	**554569**	436	Y	7	OUT	7	OUT	G
**jgi|Poptr1_1|555239|eugene3.00031872**		312	N	7	OUT	7	OUT	R
**jgi|Poptr1_1|555701|eugene3.00040414**		547	N	7	OUT	7	IN	R
**jgi|Poptr1_1|558261|eugene3.00091521**		620	N	7	OUT	9	OUT	R
**jgi|Poptr1_1|559596|eugene3.00051211**		239	N	7	IN	7	IN	R
**jgi|Poptr1_1|561026|eugene3.00060985**		377	N	8	IN	8	IN	R
**jgi|Poptr1_1|561523|eugene3.00061482**	**561523**	439	Y	7	OUT	7	OUT	G
**jgi|Poptr1_1|561994|eugene3.00061953**		642	Y	9	OUT	10	IN	G
**jgi|Poptr1_1|562471|eugene3.00070409**		334	N	6	IN	6	IN	G
**jgi|Poptr1_1|562902|eugene3.00070840**		546	N	5	OUT	7	OUT	R
**jgi|Poptr1_1|564874|eugene3.00081469**		243	N	7	IN	7	IN	R
**jgi|Poptr1_1|567196|eugene3.00101754**		483	N	7	OUT	7	IN	R
**jgi|Poptr1_1|569372|eugene3.00120025**		594	Y	9	OUT	9	OUT	G
**jgi|Poptr1_1|569632|eugene3.00120285**	**569632**	317	N	7	OUT	7	OUT	G
**jgi|Poptr1_1|571511|eugene3.00130875**		400	N	8	IN	8	IN	R
**jgi|Poptr1_1|575185|eugene3.00150580**		937	Y	8	OUT	7	OUT	R
**jgi|Poptr1_1|575407|eugene3.00150802**		283	N	7	OUT	7	OUT	R
**jgi|Poptr1_1|575408|eugene3.00150803**		283	N	7	OUT	7	OUT	R
**jgi|Poptr1_1|575753|eugene3.00151148**		345	Y	7	OUT	8	IN	R
**jgi|Poptr1_1|575848|eugene3.00151243**		296	N	7	OUT	7	OUT	R
**jgi|Poptr1_1|578289|eugene3.00180513**		639	Y	9	OUT	10	IN	G
**jgi|Poptr1_1|580779|eugene3.01230069**		492	N	8	OUT	8	OUT	R
**jgi|Poptr1_1|580803|eugene3.01230093**		296	N	7	OUT	7	OUT	R
**jgi|Poptr1_1|581554|eugene3.13140003**		381	N	7	OUT	7	OUT	R
**jgi|Poptr1_1|581829|eugene3.13340001**		313	N	8	IN	8	IN	R
**jgi|Poptr1_1|584468|eugene3.01630037**		586	Y	9	OUT	10	IN	G
**jgi|Poptr1_1|584470|eugene3.01630039**		572	N	7	OUT	7	OUT	R
**jgi|Poptr1_1|585035|eugene3.01700010**		499	N	7	OUT	9	OUT	R
**jgi|Poptr1_1|589838|eugene3.00290021**		301	N	8	IN	8	IN	R
**jgi|Poptr1_1|592088|eugene3.41270001**		283	N	7	OUT	7	OUT	R
**jgi|Poptr1_1|592680|eugene3.00440015**		313	N	8	OUT	8	OUT	R
**jgi|Poptr1_1|592879|eugene3.00440214**		592	Y	9	OUT	9	OUT	G
**jgi|Poptr1_1|593823|eugene3.00570067**		545	N	7	OUT	7	OUT	R
**jgi|Poptr1_1|594744|eugene3.06600002**		480	N	7	OUT	9	OUT	R
**jgi|Poptr1_1|594980|eugene3.00660174**		586	Y	9	OUT	9	OUT	G
**jgi|Poptr1_1|643656|grail3.0003074001**		465	N	10	OUT	10	OUT	G
**jgi|Poptr1_1|647588|grail3.0111004201**	**647588**	291	N	7	OUT	7	OUT	G
**jgi|Poptr1_1|648231|grail3.0038018602**		592	Y	9	OUT	9	OUT	G
**jgi|Poptr1_1|649249|grail3.0001031001**		491	N	10	OUT	10	OUT	G
**jgi|Poptr1_1|650244|grail3.0001115101**		405	N	8	IN	7	IN	R
**jgi|Poptr1_1|651415|grail3.0002013101**		281	N	8	IN	8	IN	R
**jgi|Poptr1_1|652845|grail3.0023006601**		279	N	8	IN	7	OUT	R
**jgi|Poptr1_1|654478|grail3.0058015501**		244	N	7	OUT	7	OUT	R

**jgi|Poptr1_1|654652|grail3.0011004001**		334	N	6	IN	6	IN	G
**jgi|Poptr1_1|656479|grail3.0010021201**		360	N	5	OUT	6	IN	G
**jgi|Poptr1_1|664019|grail3.0035021702**		424	N	7	OUT	7	OUT	R
**jgi|Poptr1_1|669814|grail3.0073010601**		248	N	8	IN	7	OUT	R
**jgi|Poptr1_1|706133|estExt_Genewise1_v1.C_LG_I1244**		328	N	7	IN	7	IN	R
**jgi|Poptr1_1|706794|estExt_Genewise1_v1.C_LG_I2590**		242	N	7	OUT	7	OUT	R
**jgi|Poptr1_1|710099|estExt_Genewise1_v1.C_LG_II0822**		242	N	7	OUT	7	OUT	R
**jgi|Poptr1_1|712123|estExt_Genewise1_v1.C_LG_III1192**		276	N	7	OUT	7	OUT	R
**jgi|Poptr1_1|720911|estExt_Genewise1_v1.C_LG_VIII2330**		466	N	7	OUT	8	OUT	R
**jgi|Poptr1_1|723021|estExt_Genewise1_v1.C_LG_IX3703**		284	N	8	IN	8	IN	R
**jgi|Poptr1_1|725778|estExt_Genewise1_v1.C_LG_X4189**		248	N	7	IN	7	IN	R
**jgi|Poptr1_1|729499|estExt_Genewise1_v1.C_LG_XIII0762**		239	N	7	OUT	7	OUT	R
**jgi|Poptr1_1|742547|estExt_Genewise1_v1.C_860119**	**742547**	402	N	7	OUT	7	OUT	G
**jgi|Poptr1_1|744336|estExt_Genewise1_v1.C_1310033**		420	N	7	OUT	7	IN	R
**jgi|Poptr1_1|755787|fgenesh4_pg.C_LG_II001967**		335	N	8	OUT	7	OUT	R
**jgi|Poptr1_1|756997|fgenesh4_pg.C_LG_III000539**		301	N	8	IN	8	IN	R
**jgi|Poptr1_1|762585|fgenesh4_pg.C_LG_VI000895**	**762585**	485	N	7	OUT	7	OUT	R
**jgi|Poptr1_1|764671|fgenesh4_pg.C_LG_VII001016**		279	N	7	OUT	7	OUT	R
**jgi|Poptr1_1|769002|fgenesh4_pg.C_LG_X000492**		342	N	7	OUT	7	OUT	R
**jgi|Poptr1_1|776100|fgenesh4_pg.C_LG_XV000528**		347	N	7	OUT	7	OUT	R
**jgi|Poptr1_1|776330|fgenesh4_pg.C_LG_XV000758**		269	N	7	OUT	7	OUT	R
**jgi|Poptr1_1|776383|fgenesh4_pg.C_LG_XV000811**		365	N	7	OUT	7	OUT	R
**jgi|Poptr1_1|796067|fgenesh4_pg.C_scaffold_21122000001**		286	N	7	IN	7	IN	R
**jgi|Poptr1_1|797267|fgenesh4_pm.C_LG_I000270**	**797267**	436	Y	7	OUT	8	IN	G
**jgi|Poptr1_1|797856|fgenesh4_pm.C_LG_I000859**		330	N	7	OUT	7	OUT	R
**jgi|Poptr1_1|799854|fgenesh4_pm.C_LG_III000319**		581	Y	7	OUT	7	OUT	R
**jgi|Poptr1_1|816796|estExt_fgenesh4_pg.C_LG_II2039**		749	N	8	IN	7	IN	R
**jgi|Poptr1_1|818178|estExt_fgenesh4_pg.C_LG_IV1437**		491	N	10	OUT	10	OUT	G
**jgi|Poptr1_1|820600|estExt_fgenesh4_pg.C_LG_VIII1017**		262	N	7	IN	7	IN	R
**jgi|Poptr1_1|820940|estExt_fgenesh4_pg.C_LG_VIII1862**	**820940**	315	N	7	OUT	7	OUT	G
**jgi|Poptr1_1|822025|estExt_fgenesh4_pg.C_LG_X0891**	**822025**	293	N	7	OUT	7	OUT	R
**jgi|Poptr1_1|827617|estExt_fgenesh4_pg.C_1070064**		547	N	7	OUT	7	OUT	R
**jgi|Poptr1_1|829403|estExt_fgenesh4_pm.C_LG_I0246**		488	N	7	OUT	9	OUT	R
**jgi|Poptr1_1|831071|estExt_fgenesh4_pm.C_LG_IV0078**		294	N	7	OUT	7	OUT	R
**jgi|Poptr1_1|831600|estExt_fgenesh4_pm.C_LG_V0513**		242	N	7	OUT	7	OUT	R
**jgi|Poptr1_1|832788|estExt_fgenesh4_pm.C_LG_VIII0619**	**832788**	284	N	7	OUT	7	OUT	R
**jgi|Poptr1_1|836846|estExt_fgenesh4_pm.C_860060**		340	N	7	OUT	8	OUT	R
**jgi|Poptr1_1|837136|estExt_fgenesh4_pm.C_1310001**		420	N	7	OUT	7	IN	R
**jgi|Poptr1_1|837467|estExt_fgenesh4_pm.C_1660030**		422	N	7	OUT	8	OUT	R
